# Penfluridol inhibits melanoma growth and metastasis through enhancing von Hippel‒Lindau tumor suppressor‐mediated cancerous inhibitor of protein phosphatase 2A (CIP2A) degradation

**DOI:** 10.1002/mco2.758

**Published:** 2024-10-13

**Authors:** Fuyan Xu, Jiao Li, Min Ai, Tingting Zhang, Yue Ming, Cong Li, Wenchen Pu, Yang Yang, Zhang Li, Yucheng Qi, Xiaomin Xu, Qingxiang Sun, Zhu Yuan, Yong Xia, Yong Peng

**Affiliations:** ^1^ Laboratory of Molecular Oncology Frontiers Science Center for Disease‐Related Molecular Network State Key Laboratory of Biotherapy West China Hospital Sichuan University Chengdu China; ^2^ Department of Biotherapy Cancer Center and State Key Laboratory of Biotherapy West China Hospital Sichuan University Chengdu China; ^3^ Rehabilitation Medicine Center State Key Laboratory of Biotherapy West China Hospital Sichuan University Chengdu China; ^4^ Frontier Medical Center Tianfu Jincheng Laboratory Chengdu China

**Keywords:** brain metastasis, cancerous inhibitor of protein phosphatase 2a, melanoma, ubiquitination, von Hippel‒Lindau tumor suppressor

## Abstract

Melanoma's high metastatic potential, especially to the brain, poses significant challenges to patient survival. The blood‒brain barrier (BBB) is a major obstacle to the effective treatment of melanoma brain metastases. We screened antipsychotic drugs capable of crossing the BBB and identified penfluridol (PF) as the most active candidate. PF reduced melanoma cell viability and induced apoptosis. In animal models, PF effectively inhibited melanoma growth and metastasis to the lung and brain. Using immunoprecipitation combined with high‐resolution mass spectrometry, and other techniques such as drug affinity responsive target stability, we identified CIP2A as a direct binding protein of PF. CIP2A is highly expressed in melanoma and its metastases, and is linked to poor prognosis. PF can restore Protein Phosphatase 2A activity by promoting CIP2A degradation, thereby inhibiting several key oncogenic pathways, including AKT and c‐Myc. Additionally, von Hippel‒Lindau (VHL) is the endogenous E3 ligase for CIP2A, and PF enhances the interaction between VHL and CIP2A, promoting the ubiquitin‒proteasome degradation of CIP2A, thereby inhibiting melanoma growth and metastasis. Overall, this study not only suggests PF's potential in treating melanoma and its brain metastases but also highlights CIP2A degradation as a therapeutic strategy for melanoma.

## Graphical abstract

This study identifies penfluridol (PF) as a promising candidate for treating melanoma, particularly its brain metastases, by targeting and degrading CIP2A via the ubiquitin‒proteasome pathway. PF restores Protein Phosphatase 2A activity, inhibits key oncogenic pathways, and enhances the interaction between E3 ligase von Hippel‒Lindau and CIP2A, leading to reduced melanoma growth and metastasis in animal models.

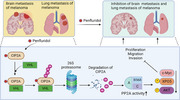

## 1 INTRODUCTION

Advancements in targeted therapies and immunotherapies have notably decreased melanoma mortality rates. However, patients with metastatic melanoma, especially those with brain metastases, continue to face poor prognoses.^1^ Melanoma has the highest incidence of brain metastasis among all cancers.^2^ Improved diagnostic techniques and better control of extracranial cancer have increased the occurrence of melanoma brain metastasis (MBM). Brain metastasis often manifests as neurological impairment, indicating poor quality of life and limited survival. One significant challenge in treating brain metastases is the blood‒brain barrier (BBB), which limits the effectiveness of many systemic therapies.^3^ Another major obstacle in treating brain metastases is that many clinical trials exclude those patients, leading to a lack of new treatment options.^4^ Lung metastasis is another frequent and severe threat to the survival of melanoma patients.^5^ As such, there is an urgent need for new and effective anticancer drugs that can rapidly translate to clinical use, especially those capable of crossing the BBB.

Novel drug development faces increasing challenges such as increasing development costs, high failure rates, and long development cycles. Drug repositioning is an important strategy to improve the efficiency of anticancer drug discovery. Due to known safety profiles, pharmacokinetics, and mechanisms of activities, once their efficacy in new indications is established, these drugs can quickly advance to phase II and III clinical trials for clinical translation.^6^ There have been several successful examples in this area. For instance, thalidomide, originally used for morning sickness, is now used to treat multiple myeloma,^7^ and the immunosuppressant rapamycin is used for anticancer therapy.^8^ Previous studies have shown that patients taking antipsychotic drugs capable of crossing the BBB have a lower incidence of cancer, indicating the potential of these drugs in cancer treatment.^9^, ^10^ For example, chlorpromazine has shown potential in inhibiting oral cancer,^11^ and trifluoperazine has demonstrated inhibitory effects on breast cancer.^12^ Notably, trifluoperazine and fluphenazine have also shown efficacies in treating brain and lung metastases of cancers.^12^, ^13^ These findings suggest that further research into the anticancer efficacies and mechanisms of antipsychotic drugs might provide new perspectives for cancer treatment.

Penfluridol (PF) is another antipsychotic drug for treating schizophrenia and acute psychosis. Recent studies have shown that it exhibits promising anticancer potentials. For instance, it suppresses the progression of esophageal squamous cell carcinoma by regulating the AMPK/FOXO3a/BIM signaling, inhibiting glycolysis, and promoting apoptosis.^14^ In non‐small cell lung cancer, it blocks autophagic flux and induces autophagosome accumulation, leading to ATP depletion and non‐apoptotic cell death.^15^ In pancreatic cancer, it reduced cancer cell proliferation by modulating the prolactin receptor.^16^ Additionally, PF downregulates the levels of integrin α6 and β4, inhibits cell survival and migration, and induces apoptosis, thereby suppressing the growth and brain metastasis of breast cancer.^17^ These studies indicate that PF has broad therapeutic potential in cancer by various mechanisms. However, the efficacy of PF against melanoma growth and metastasis (including brain and lung metastases) remains unclear. Notably, only a limited number of previous studies have identified the direct target of PF to treat cancer, limiting the in‐depth elucidation of its anticancer mechanism and further development.

CIP2A is a protein encoded by the KIAA1524 gene located on human chromosome 3q13.13.^18^ Comprising 905 amino acids, CIP2A features an N‐terminal armadillo repeat domain (amino acids 1−560) and a C‐terminal coiled‐coil region.^19^ As an endogenous inhibitor of PP2A, CIP2A interacts with the B56α and B56γ subunits of PP2A, forming a CIP2A‐B56α‐PP2Ac pseudo‐trimer, thereby inhibiting PP2A's tumor‐suppressive functions.^20^ This mechanism involves CIP2A binding to the B56 subunit of PP2A through its conserved N‐terminal region, promoting CIP2A homodimerization and blocking PP2A‐A subunit binding. Simultaneously, CIP2A obstructs the LxxIxE motif substrate binding site on B56α, further inhibiting PP2A by competing with B56α substrates.^21^, ^22^

CIP2A is overexpressed in more than 70% of tumors, which correlates with proliferation, migration, invasion, and treatment resistance in cancer.^23^, ^24^ It enhances c‐Myc protein stability by inhibiting PP2A‐mediated dephosphorylation of c‐Myc S62.^25^ It also activates several other signaling pathways, such as PI3K/Akt/mTOR, Wnt/β‐catenin, and Ras‐Raf‐MEK‐ERK, promoting cancer cell proliferation and survival while inhibiting apoptosis.^23^, ^24^ Furthermore, CIP2A regulates the cell cycle and mitosis by interacting with proteins such as Plk1, E2F1, Akt, and DAPK1.^23^, ^24^ CIP2A overexpression in melanoma correlates with poor prognosis, underscoring its potential as a therapeutic target for the disease.^26^, ^27^

Reducing the expression of CIP2A has demonstrated the ability to decrease cell proliferation, invasion, and migration, while increasing apoptosis in melanoma.^23^, ^28^ Protein degradation within cells maintains dynamic equilibrium by breaking down damaged or unneeded proteins for recycling or disposal.^29^ Major pathways for intracellular protein degradation include the lysosomal pathway and the ubiquitin‒proteasome system (UPS).^30^ UPS involves covalently attaching ubiquitin molecules to target proteins, marking them for degradation by the proteasome. E3 ligases play a key role in UPS by recognizing specific target proteins and transferring ubiquitin molecules to them.^31^ Based on the UPS, strategies such as molecular glues and proteolysis‐targeting chimeras (PROTACs) have been developed and they have shown promising anticancer potentials.^32^ Several compounds, including metformin, bortezomib, and erlotinib, exert anticancer effects by inducing degradation of CIP2A.^33^, ^34^, ^35^ This provides a basis for developing novel anticancer drugs targeting CIP2A degradation, thereby opening promising avenues for treating melanoma and its metastases.

In this study, we screened various antipsychotic drugs for anti‐melanoma activity and found that PF significantly inhibits melanoma growth, brain metastasis, and lung metastasis. We confirmed that CIP2A is the direct binding protein of PF and elucidated the mechanism by which PF reduces CIP2A levels in melanoma. This study provides preliminary experimental evidence for the potential application of PF in treating melanoma and its brain metastases and offers insights for developing new anti‐melanoma drugs based on CIP2A‐targeted degradation.

## 2 RESULTS

### 2.1 The antipsychotic drug PF inhibits melanoma growth

**FIGURE 1:**
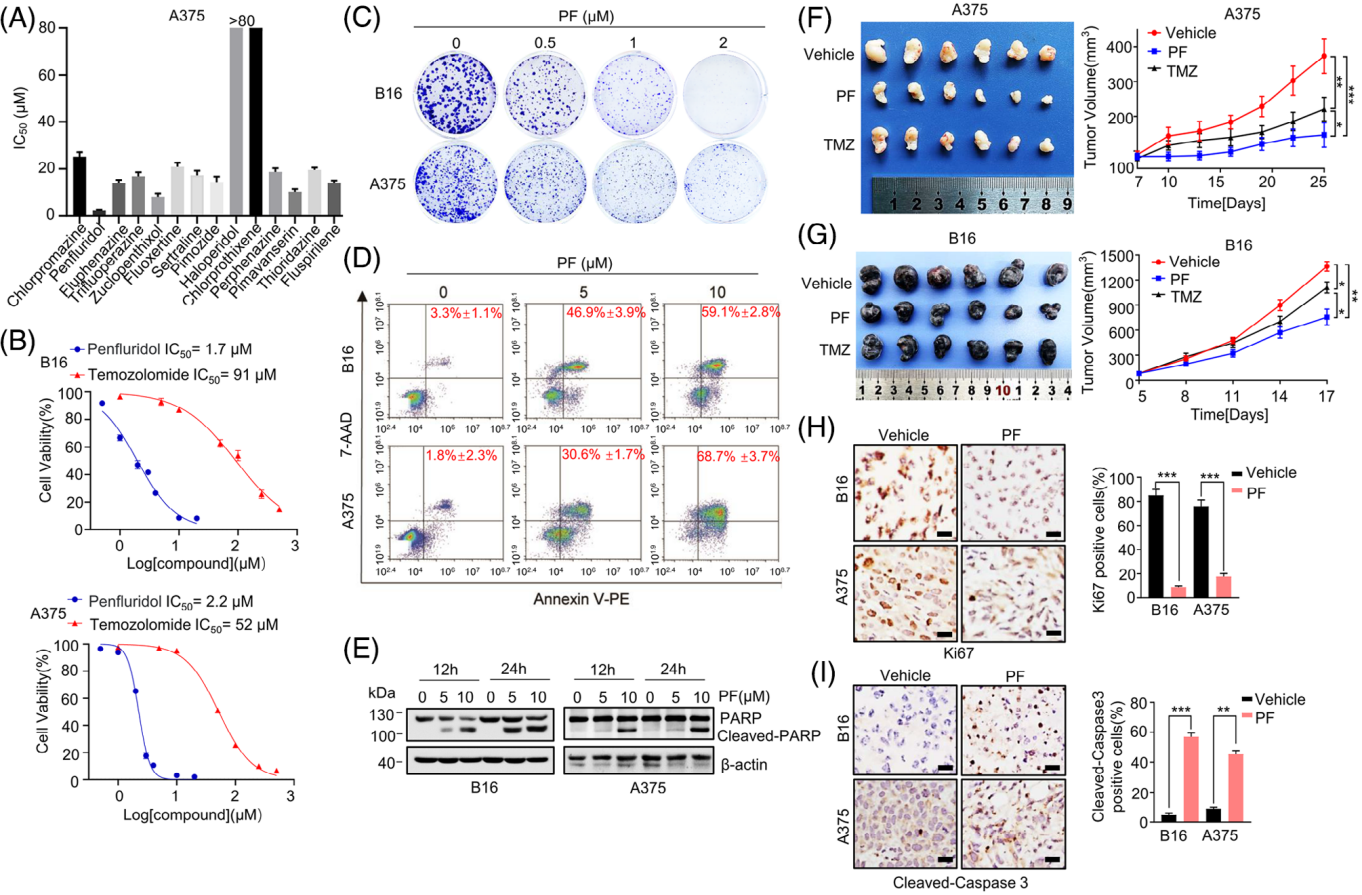
Penfluridol (PF) inhibits melanoma cell proliferation in vitro and tumor growth in vivo. (A) IC_50_ values (µM) of 14 antipsychotic drugs against A375 cell after 72 h of treatment. (B) B16 and A375 cells treated with various concentrations of PF and temozolomide (TMZ) for 72 h and MTT assay was used to detect the inhibitory effects. (C) Colony formation assay was used to detect the effect of PF on melanoma cell proliferation after treatment for 10 days. (D) After treating B16 and A375 cells with different concentrations of PF for 24 h, Annexin V‐PE/7‐AAD staining was performed, followed by flow cytometry to assess the effect of PF on cell apoptosis. (E) The expression levels of PARP and cleaved PARP were detected in B16 and A375 cells treated with various concentrations PF for 12 and 24 h. The above experiments were performed in triplicate. (F and G) Left: representative images of isolated tumors from mice inoculated with B16 and A375 subcutaneous tumors, treated with vehicle, PF (15 mg/kg), and TMZ (40 mg/kg). Treatments were administered twice a week via intraperitoneal injection (*n* = 6). Right: tumor volume changes over time in the two tumor models. (H and I) Immunohistochemical staining was performed on paraffin‐embedded B16 and A375 tumor tissues from mice using anti‐Ki67 and anti‐cleaved‐Caspase 3 antibodies. In the images, brown staining indicates positively stained cells. The scale bars represent 50 µm (*n* = 3). Data are expressed means ± standard deviation (SD). ^*^ *p* < 0.05, ^**^ *p* < 0.01, ^***^ *p* < 0.001.Poly (ADP‐ribose) polymerase, PARP; 3‐(4,5‐Dimethylthiazol‐2‐yl)‐2,5‐Diphenyltetrazolium Bromide, MTT.

To identify candidate drugs for inhibiting melanoma and its brain metastases from marketed antipsychotic drugs capable of crossing the BBB, we treated human A375 melanoma cells with 14 approved antipsychotic drugs for 72 h and measured cell viability using MTT (3‐(4,5‐Dimethylthiazol‐2‐yl)‐2,5‐Diphenyltetrazolium Bromide)assay. As shown in Figure 1A, the first‐generation diphenylbutylpiperidine antipsychotic drug PF exhibited the highest inhibitory activity.

To further investigate PF's inhibitory activity on melanoma, we included six other melanoma cell lines (B16, A875, HT144, C32, WM115, A2058). The results indicated that PF significantly inhibited the viability of all melanoma cell lines, with IC_50_ values below 5 µM at 72 h (Figure S1A). However, PF's inhibitory activity on normal mammary epithelial cells (MCF‐10A) was at least five times less potent than on melanoma cells (Figure S1A), suggesting that PF may selectively target cancer cells.

Both murine melanoma B16 cell line and human A375 melanoma cell line are extensively used in studies of melanoma growth and metastasis. Therefore, they were primarily used in subsequent experiments. Temozolomide (TMZ) is a first‐line treatment for melanoma with brain metastasis. Our results showed that PF had superior inhibitory activity on melanoma growth compared to TMZ in both B16 and A375 cells (Figure 1B), with similar results observed in colony formation assays (Figures 1C and S1B). Moreover, PF induced apoptosis in melanoma cells in a dose‐dependent manner (Figures 1D and S1C) by increasing levels of cleaved PARP (Poly (ADP‐ribose) polymerase), a hallmark of apoptosis (Figure 1E).

Next, we evaluated the potential of PF to inhibit melanoma growth in vivo. Subcutaneous tumor models were established using B16 and A375 cells, respectively, followed by treatment with PF (15 mg/kg) or TMZ (40 mg/kg) twice a week. PF inhibited the growth of human A375 tumors by 61%, compared to 41% for TMZ (Figure 1F). In the B16 murine model, tumor volume was reduced by 45% in the PF‐treated group, compared to a 21% reduction in the TMZ group (Figure 1G). These results indicate that PF has better inhibitory activity on melanoma in mice over TMZ. Immunohistochemical staining of Ki‐67 and cleaved‐Caspase‐3 in mouse tumors revealed that PF induced apoptosis and inhibited proliferation in vivo (Figure 1H,I).

During the treatment, the body weights of the mice did not show significant change during treatment (Figure S1D). The general health of the mice, including their behaviors, was also monitored, and no significant abnormalities were observed in the PF treated groups. Preliminary toxicity tests were conducted on healthy C57BL/6 mice without tumor implantation. Hematoxylin and eosin (H&E) staining of major organs indicated no obvious damage to the heart, liver, spleen, lungs, and kidneys in PF‐treated mice (Figure S1E). These data suggest that PF has a good preliminary safety profile at effective anti‐melanoma doses in mice.

### 2.2 PF inhibits migration and invasion of melanoma cells in vitro and showed efficacies in experimental brain and lung metastasis models of melanoma in vivo

**FIGURE 2:**
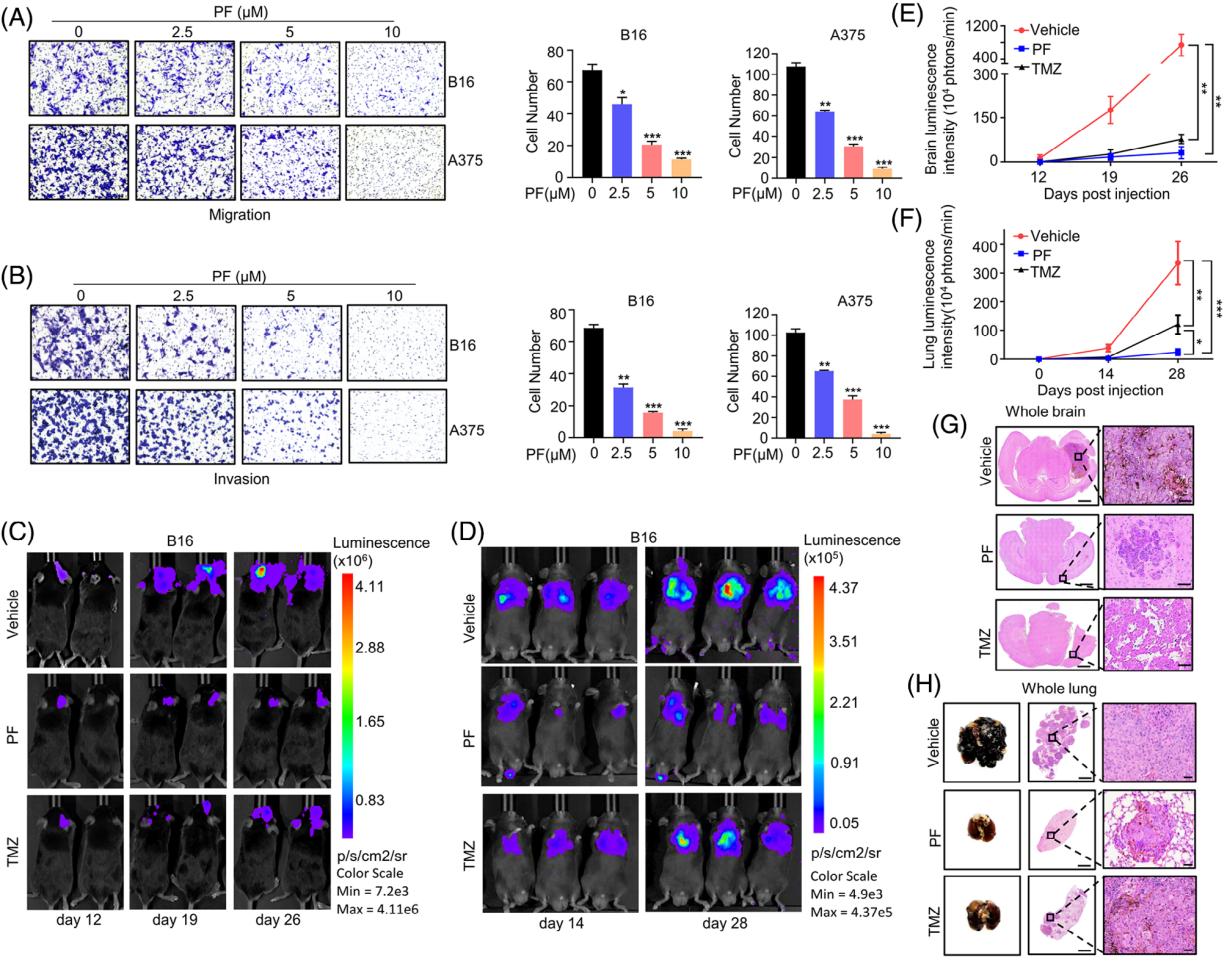
Penfluridol (PF) inhibits migration and invasion of melanoma cells in vitro and suppresses brain and lung metastasis in vivo. After treatment with various concentrations of PF for 24 h. Transwell migration (A) and invasion (B) assays were performed in B16 and A375 cells. This experiment was performed in triplicate. The experimental brain metastasis (C) and lung metastasis (D) models were established using B16 cells. Seven days later, treatments with PF (15 mg/kg) and temozolomide (TMZ) (40 mg/kg) were administered twice a week. (C and D) Representative bioluminescence images at the indicated time points (*n* = 6). (E and F) Quantitative analysis of the signal intensity in the images from (C) and (D). (G and H) Representative hematoxylin and eosin (H&E) staining images of brain and lung metastatic lesions in mice from (C) and (D). Scale bar: 1 mm (left) and 50 µm (right). Data are presented as means ± standard deviation (SD). ^*^ *p* < 0.05, ^**^ *p* < 0.01, ^***^ *p* < 0.001.

To investigate the inhibitory potential of PF on melanoma metastasis, we first conducted Transwell assays. Compared to the control group, the migration and invasion abilities of B16 and A375 cells in the PF‐treated group were significantly reduced in a dose‐dependent manner (Figure 2A,B). We also employed a migration assay to compare the effects of PF and TMZ (Figure S1F), demonstrating that PF exhibited superior inhibitory activity on melanoma migration compared to TMZ in both B16 and A375 cells.

Next, we established an experimental model of MBM by injecting B16Br‐Luc cells into the intracarotid artery of C57BL/6 mice. Drug treatment began 1 week after cell injection, following the same regimen as in the above subcutaneous tumor model. In vivo imaging results showed rapid growth of intracranial melanoma, while PF greatly inhibited brain metastasis growth and exhibited a slightly better activity than TMZ (Figure 2C,E,G).

Given that melanoma is also prone to lung metastasis, we established an experimental lung metastasis model by injecting B16‐Luc cells into C57BL/6 mice via the tail vein. The data indicated that PF significantly inhibited lung metastasis growth, outperforming TMZ (Figure 2D,F,H). In summary, our data show that PF has the potential to inhibit both brain and lung metastasis of melanoma.

### 2.3 CIP2A is the target protein of PF

**FIGURE 3:**
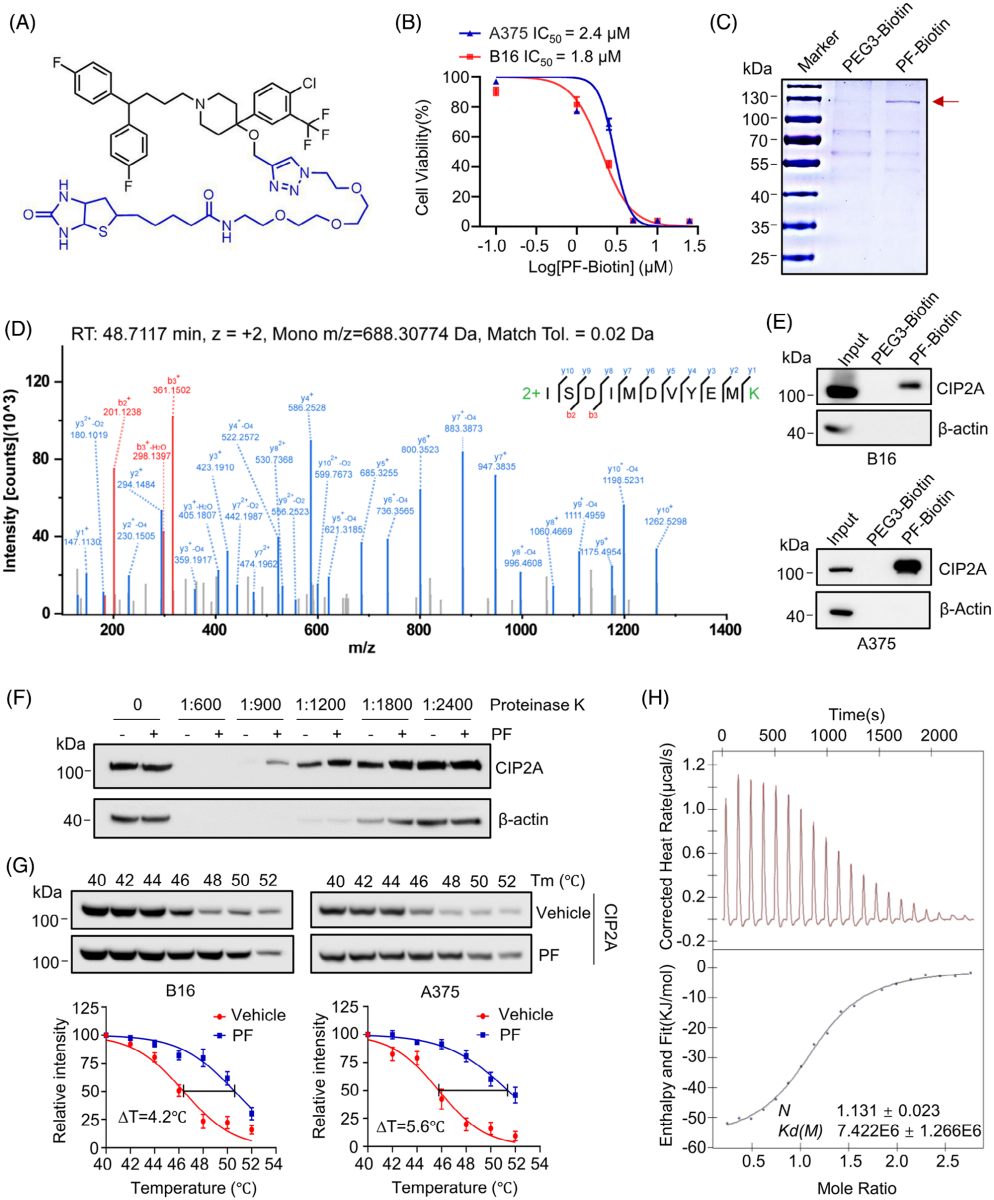
Penfluridol (PF) directly binds to CIP2A in melanoma cells. (A) Chemical structure of PF‐Biotin. (B) The inhibitory effects of PF‐Biotin on the proliferation of B16 and A375 cells after treatment for 72 h. (C) After treating A375 cells with PF‐Biotin (1 µM) or PEG3‐Biotin (1 µM) for 12 h, proteins were extracted for streptavidin‒biotin‐based immunoprecipitation. The gel was stained with Coomassie blue after SDS‐3‐(4,5‐Dimethylthiazol‐2‐yl)‐2,5‐Diphenyltetrazolium Bromide, MTT. PAGE separation, with the red arrow indicating the excised band. (D) The excised bands were processed and subjected to high‐resolution mass spectrometry for qualitative analysis. The figure shows the mass spectrum of a specific peptide segment of CIP2A. (E) Streptavidin‒biotin immunoprecipitation was performed on the protein mixture extracted from melanoma cells. Subsequent immunoblot assays analysis confirmed that CIP2A is a binding protein of PF. (F) Drug affinity responsive target stability (DARTS) assay measured the impact of PF on the proteolytic stability of CIP2A. After treating A375 cells with PF, whole‐cell lysates were incubated with different concentrations of proteinase K for 5 min, and then the amounts of CIP2A and reference proteins in these samples were detected by immunoblot assays. (G) Cellular thermal shift assay (CETSA) was performed to assess the effect of PF on the thermal stability of CIP2A. Following PF treatment of melanoma cells, the levels of non‐denatured CIP2A protein were measured at different temperature gradients, and protein band intensities were quantitatively analyzed using ImageJ. (H) Isothermal titration calorimetry (ITC) analysis of PF binding to recombinant CIP2A (1‐560) protein. PF dissolved in a specific solution was titrated into 50 µM recombinant Hu‐CIP2A (1‐560) protein. The data were fitted using the one‐site binding model. Sodium dodecyl‐sulfate polyacrylamide gel electrophoresis, SDS‐PAGE.

To precisely explore the biological mechanism through which PF exerts its anti‐melanoma activity, we synthesized a biotin‐labeled PF probe, PF‐Biotin (Figure 3A). In vitro anti‐melanoma efficacies of PF‐Biotin in B16 and A375 cells indicated that the inclusion of biotin did not significantly affect PF's inhibitory activities against melanoma (Figures 1B and 3B). Subsequently, we employed streptavidin‒biotin immunoprecipitation combined with high‐resolution mass spectrometry to identify PF's binding proteins by using PEG‐Biotin as the negative control. Compared to the PEG3‐Biotin, a specific band between 100 and 130 kDa was captured by PF‐Biotin (Figure 3C). Mass spectrometry analysis identified that CIP2A is in the specific band, with the highest scores and top abundance (Figure 3D and Table S1). Immunoblotting following biotin‐immunoprecipitation in B16 and A375 cells further confirmed the interaction between PF and CIP2A (Figure 3E).

We further used drug affinity responsive target stability assay to investigate whether CIP2A is the target of PF. The Coomassie Brilliant Blue staining results showed that a specific protein band appeared at around 100 kDa in the PF‐treated sample upon the enzymatic hydrolysis of proteinase K at a dilution ratio of 1:900 (as indicated by the red circular symbol in Figure S2). The position of this band is consistent with the molecular weight of CIP2A protein. Subsequently, we performed Western blot analysis on the above‐treated samples and the data indicated that that PF protects CIP2A from proteinase K‐induced protein hydrolysis (Figure 3F). The cellular thermal shift assay demonstrated that CIP2A was stabilized against thermal changes under PF treatment (Figure 3G). These assays strongly suggested that PF binds to CIP2A. Additionally, we purified the recombinant CIP2A protein (Figure S3) performed isothermal titration calorimetry assay to determine whether PF directly interacts with CIP2A. A strong binding affinity between PF and CIP2A protein was observed, with a dissociation constant of 7.42 µM (Figure 3H). In summary, our results strongly suggest that CIP2A is a direct binding protein of PF.

### 2.4 PF interacts with CIP2A to exert anti‐melanoma activity

**FIGURE 4:**
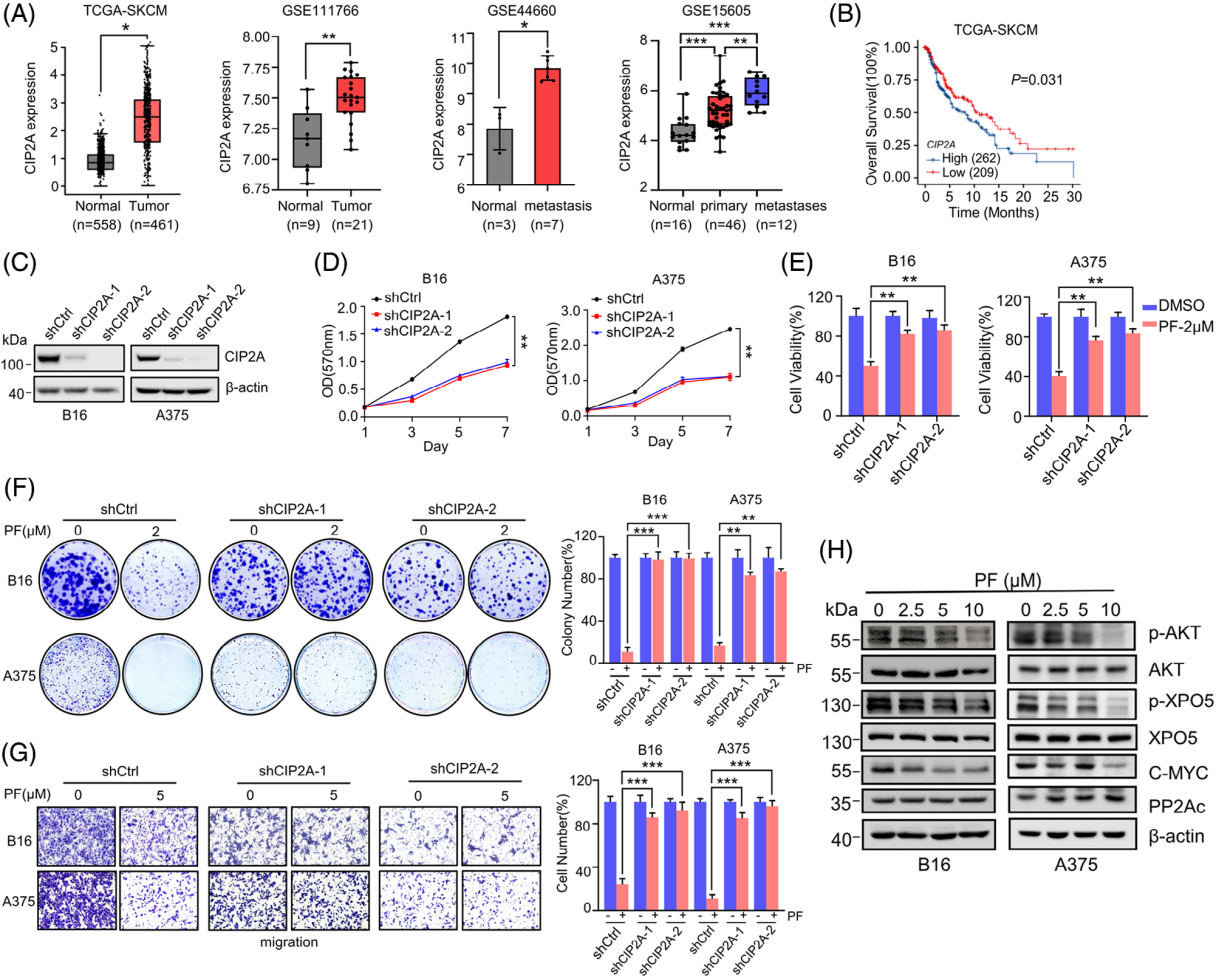
Penfluridol (PF) inhibits melanoma cells growth and metastasis in a CIP2A‐dependent manner. (A) CIP2A is highly expressed in melanoma and is associated with poor prognosis in patients. CIP2A mRNA levels were detected in melanoma samples, normal nevus samples, and melanoma metastasis samples. A cDNA microarray dataset from The Cancer Genome Atlas (TCGA) and three Gene Expression Omnibus (GEO) cDNA microarray datasets (GSE111766, GSE44660, and GSE15605) with clinical annotation were analyzed. (B) Kaplan‒Meier survival analysis based on the different expression of CIP2A mRNA in the TCGA‐SKCM dataset. (C) The knockdown efficiency of CIP2A by shRNA in B16 and A375 cells was detected by immunoblot assays. (D) The impact of CIP2A knockdown on the proliferation of melanoma cells in the MTT assay. (E) Changes in sensitivity to PF treatment in melanoma cells after CIP2A knockdown in the MTT assay. (F) After treating B16 and A375 cells with CIP2A knockdown with PF (2 µM) for 10 days, the effect of CIP2A knockdown on PF's inhibition on melanoma cell growth was assessed using colony formation counting. (G) Knockdown of CIP2A reduces the inhibitory activity of PF on melanoma cells migration. The cells were treated with 5 µM PF for 24 h, and migration of the cells were detected with Transwell assay. For panels (E‒G), the results are normalized to the untreated conditions (Dimethylsulfoxide, DMSO) in each cell line. (H) After treating B16 and A375 cells with different concentrations of PF for 24 h, cell lysates were collected for immunoblot assays analysis to detect the levels of CIP2A downstream‐related proteins. ^*^ *p* < 0.05, ^**^ *p *< 0.01, ^***^ *p* < 0.001.

We hypothesized that PF might inhibit melanoma by regulating CIP2A. First, we analyzed the expression levels of CIP2A in normal nevus samples and melanoma in The Cancer Genome Atlas (TCGA) and found that its mRNA was upregulated in melanoma (Figure 4A). We then extracted and analyzed multiple datasets from the Gene Expression Omnibus (GEO) database. Compared to normal nevi tissues, CIP2A is upregulated in melanoma and more prominent in metastatic melanoma (Figure 4A). To understand the correlation between CIP2A expression and melanoma patient survival, we conducted Kaplan‒Meier survival analysis using GEPIA on data from TCGA and BEST (https://rookieutopia.com/). High CIP2A expression was associated with reduced patient survival (Figures 4B and S4). Therefore, these data indicate that CIP2A expression is closely related to melanoma progression, suggesting its oncogenic role in melanoma.

Next, we adopted a loss‐of‐function strategy to knockdown CIP2A expression in melanoma cells to understand its role in role in melanoma progression and in PF's anti‐melanoma activity (Figure 4C). As expected, CIP2A knockdown significantly reduced the proliferation of B16 and A375 cells (Figure 4D). Moreover, knockdown of CIP2A markedly decreased melanoma cells' sensitivity to PF (Figure 4E,F). Similarly, Transwell assays showed that reduced CIP2A levels diminished the sensitivity of cells to PF treatment (Figure 4G), indicating that PF exerts its anti‐melanoma role in a CIP2A‐dependant manner. To further confirm this, we re‐expressed CIP2A in B16 and A375 cells with CIP2A knockdown (Figure S5A). Restoration of CIP2A levels significantly increased the proliferation of melanoma cells (Figure S5B) and rescued the inhibitory activity of PF on the growth of these cells (Figure S5C,D). Similarly, Transwell assays showed that rescue of CIP2A reversed the reduction in migration inhibition by PF in CIP2A knockdown cells (Figure S5E). These data further demonstrate that the anti‐proliferative and anti‐metastatic effects of PF are mediated by CIP2A in melanoma cells.

CIP2A exerts its oncogenic role through various pathways by inhibiting PP2A enzymatic activity.^28^ CIP2A prevents the degradation of c‐Myc by inhibiting PP2A‐mediated dephosphorylation of c‐Myc at S62, thereby stabilizing c‐Myc protein.^36^ The PP2A complex reduces Akt activity by promoting dephosphorylation of Akt at Thr308 and Ser473.^37^ Our previous research found that exportin‐5 (XPO5) is also a substrate of PP2A, which inhibits XPO5 phosphorylation at Ser416, thereby regulating XPO5's pre‐miRNA transport capacity and promoting miRNA expression to modulate liver cancer progression.^38^ To explore PF's impact on CIP2A downstream pathway, we treated B16 and A375 cells with different concentrations of PF and found no significant change in the protein levels of the PP2A catalytic subunit PP2Ac. The levels of p‐AKT (Ser473) and p‐XPO5 (Ser416) decreased with increasing PF concentration; c‐Myc protein levels also decreased after PF treatment (Figure 4H). In summary, these data suggest that PF may restore the anticancer activity of PP2A by inhibiting CIP2A, thereby suppressing multiple oncogenic signaling pathways and inhibiting melanoma growth and metastasis.

### 2.5 PF promotes CIP2A degradation through the ubiquitin‒proteasome system pathway

**FIGURE 5:**
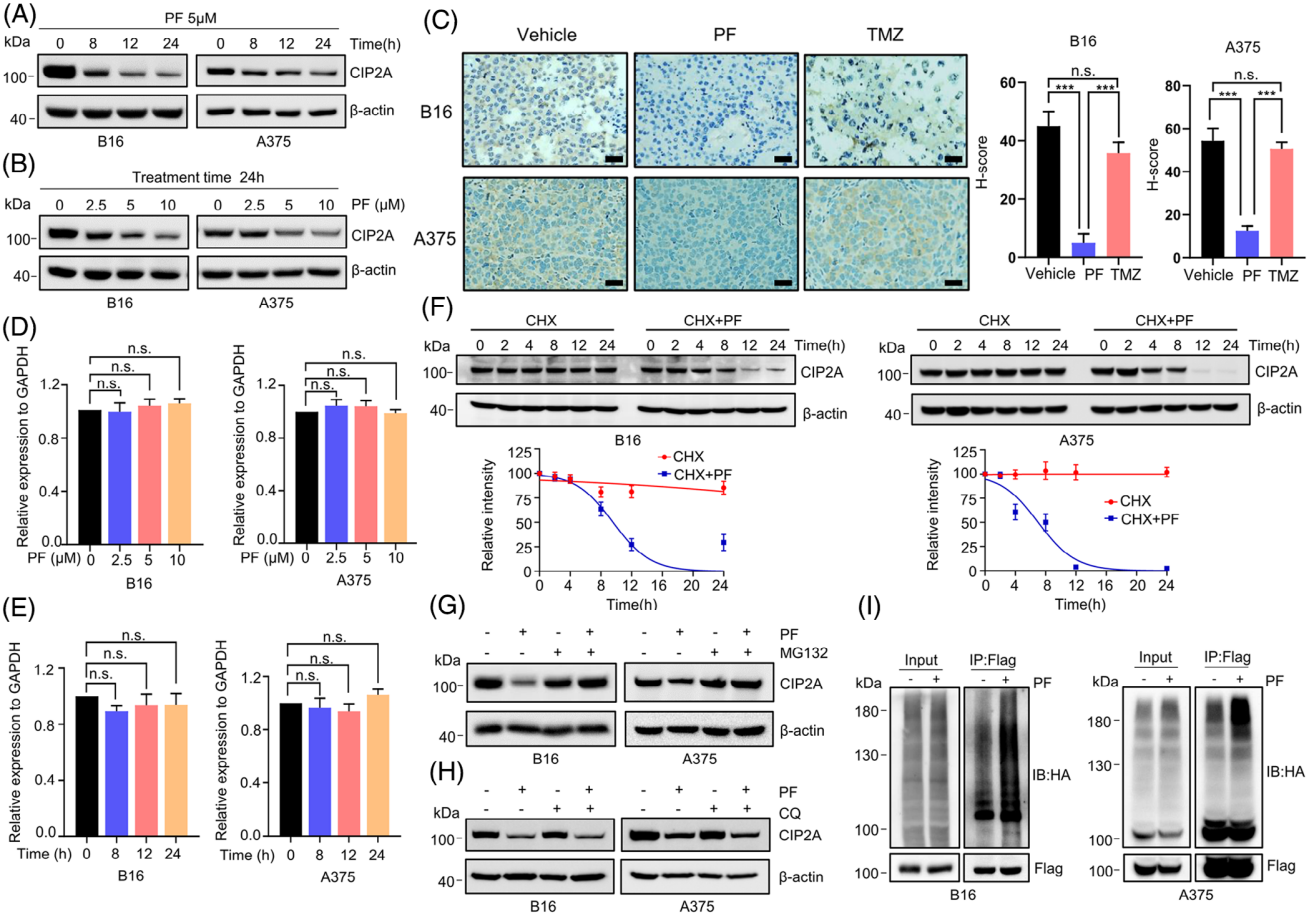
Penfluridol (PF) promotes CIP2A degradation through ubiquitin‒proteasome pathway. (A) The effects of 5 µM PF on protein levels of CIP2A in B16 and A375 cells after treatment for different time. (B) After treating B16 and A375 cells with different concentrations of PF for 24 h, protein levels of CIP2A were analyzed. (C) Representative immunohistochemistry (IHC) staining of CIP2A protein levels in subcutaneous tumors were treated with PF and temozolomide (TMZ). Quantitative analysis of the data is shown on the right. Scale bar: 50 µm. (D) After treating B16 and A375 cells with different concentrations of PF for 24 h, mRNA levels of CIP2A were analyzed by RT‐qPCR. (E) After treating B16 and A375 cells with 5 µM PF for different time, mRNA levels of CIP2A were analyzed by RT‐qPCR. (F) Effect of PF on CIP2A protein stability. B16 and A375 cells were treated with 50 µM cycloheximide (CHX) in the absence or presence of 5 µM PF for indicated time points, and the changes in CIP2A protein levels over time were analyzed. Quantitative analysis of the protein levels is also shown. (G) The effect of PF (5 µM), MG132 (20 µM), and their combination on CIP2A protein levels in B16 and A375 cells. The cells were treated with PF for 18 h, followed by MG132 for an additional 6 h in the combination treatment group. (H) The effect of PF (5 µM), chloroquine (CQ) (50 µM), and their combination on CIP2A protein levels in B16 and A375 cells. The cells were pre‐treated with CQ for 2 h, then PF was added for a further 24 h. (I) B16 and A375 cell stably expressing Flag‐CIP2A were transfected with an HA‐tagged ubiquitin expression plasmid (HA‐Ub). Then, the cells were treated with 5 µM PF overnight. Subsequently, 20 µM MG132 was added for an additional 6 h. Immunoprecipitation was performed using an anti‐Flag antibody, followed by analysis of CIP2A ubiquitination using an anti‐HA antibody. The grayscale of the protein bands was quantified using ImageJ. Data are presented as means ± standard deviation (SD). n.s., not significant; ^***^ *p *< 0.001.

To investigate how PF modulate CIP2A, we firstly examined the effect of PF on CIP2A protein levels. After treatment with 5 µM PF for varying time, CIP2A protein levels in B16 and A375 cells gradually decreased in a time‐dependent manner (Figure 5A). Further studies demonstrated that PF dose dependently induces CIP2A downregulation (Figure 5B). Additionally, immunohistochemical staining of B16 and A375 subcutaneous tumor tissues showed a significant reduction in CIP2A protein levels in the PF‐treated groups (Figure 5C).

PF may downregulate CIP2A protein levels at transcriptional, post‐transcriptional, translational, or post‐translational levels. To analyze the mechanism of CIP2A downregulation, we first assessed the effect of PF on CIP2A mRNA levels. After treating melanoma cells with different concentrations of PF for 24 h, no significant impact of PF on the mRNA levels was observed (Figure 5D). We then also used 5 µM PF treated B16 and A375 cells for varying time, CIP2A mRNA levels did not change (Figure 5E). We then hypothesized that PF might affect CIP2A protein stability. To test this possibility, we used the protein synthesis inhibitor cycloheximide (CHX) to block protein synthesis. CIP2A levels did not significantly decrease after CHX treatment. However, in cells co‐incubated with CHX or PF plus CHX, CIP2A began to downregulate after 4 h (Figure 5F). Obviously, PF significantly shortened the half‐life of CIP2A protein in melanoma cells, affecting its stability.

According to previous reports, CIP2A degradation may occur through both the lysosomal and UPS pathways.^39^, ^40^ To determine which pathway PF influences CIP2A degradation, we first used the classic proteasome inhibitor MG‐132 in vitro to see if it could reverse PF‐induced CIP2A degradation and the data confirmed this reversion (Figure 5G). However, the lysosomal inhibitor chloroquine did not prevent CIP2A degradation (Figure 5H). Furthermore, PF treatment obviously induced CIP2A polyubiquitination in melanoma cells stably expressing Flag‐CIP2A (Figure 5I). In summary, these data indicate that PF probably promotes CIP2A degradation in melanoma through the UPS pathway.

### 2.6 PF enhances the interaction of CIP2A with the E3 ligase von Hippel‒Lindau to promote CIP2A degradation

**FIGURE 6:**
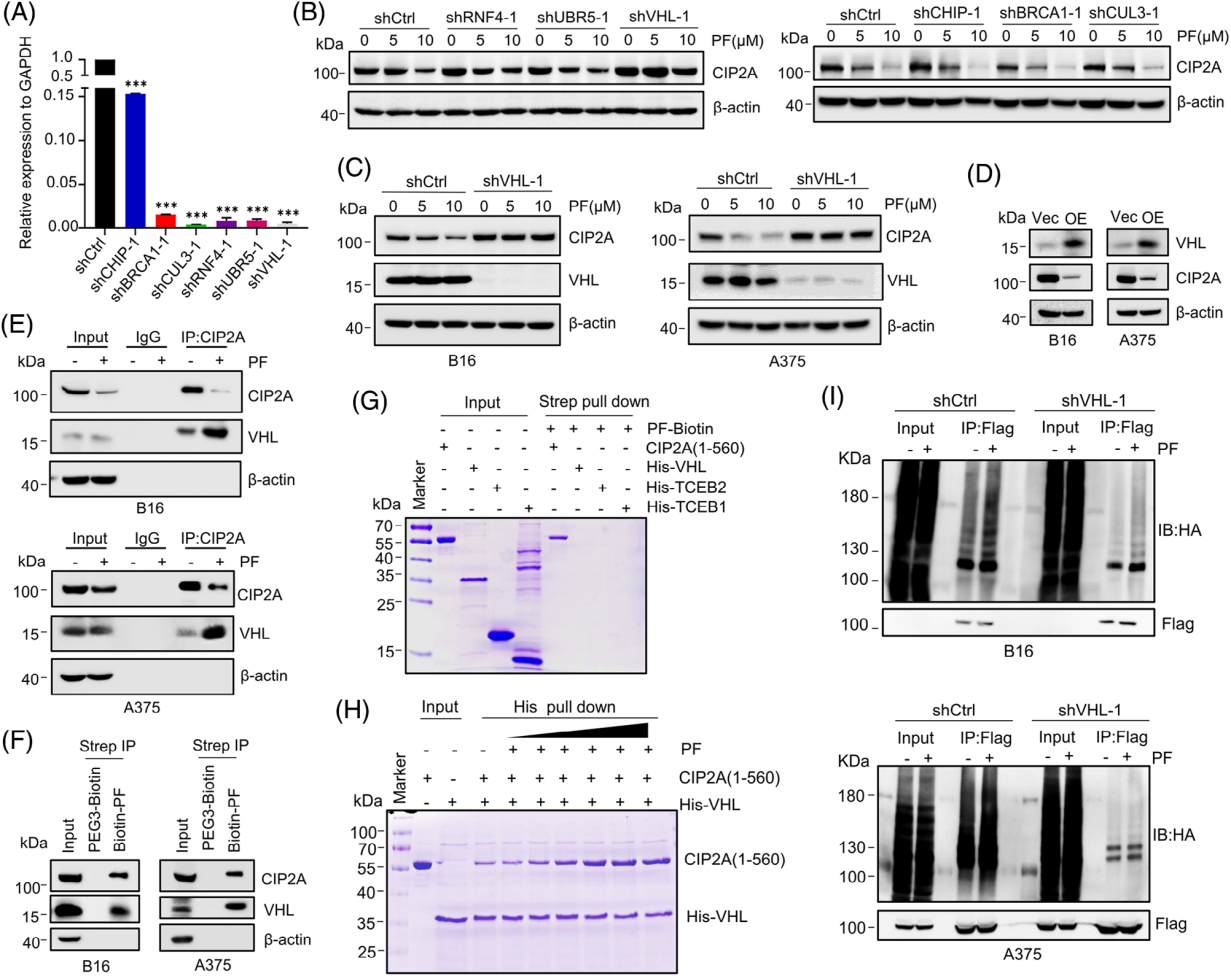
Penfluridol (PF) promotes CIP2A degradation by enhancing the interaction between E3 ligase von Hippel‒Lindau (VHL) and CIP2A. (A) Detection of knockdown efficiency of shRNA targeting BRCA1, CUL3, RNF4, UBR5, CHIP, and VHL E3 ligases in A375 cells. The mRNA levels of the stable transfected cells were detected with RT‐qPCR. Data are presented as mean ± standard deviation. ^***^ *p* < 0.001. (B) The effect of PF on CIP2A protein levels in A375 cells with stable knockdown of BRCA1, CUL3, RNF4, UBR5, CHIP, and VHL was analyzed after treatment for 24 h. (C) The ability of PF to downregulate CIP2A protein levels was inhibited in A375 and B16 cells with stable knockdown of VHL. (D) The protein levels of VHL and CIP2A in A375 and B16 cells overexpressing VHL. OE represents cells overexpressing VHL. (E) After treating B16 and A375 cells with 5 µM PF for 24 h, immunoprecipitation was performed using an anti‐CIP2A antibody. The effect of PF on the interaction between CIP2A and VHL in cells was then detected by immunoblot assays. (F) After PF‐Biotin treatment of B16 and A375 cells, a streptavidin‒biotin pull‐down assay was performed. Subsequently, immunoblot assays analysis was conducted to detect the interaction between PF‐Biotin and CIP2A and VHL in the cells. (G) After incubating recombinant CIP2A protein (1‐560), His‐VCB, and PF‐Biotin in vitro, a streptavidin‒biotin pull‐down assay was performed. The binding of PF‐Biotin to the various purified proteins was then detected by Coomassie blue staining. (H) After incubating recombinant CIP2A protein (1‐560), His‐VHL, and different concentrations of PF in vitro, His pull‐down assay was conducted. The effect of PF on the interaction between His‐VHL and CIP2A protein was subsequently detected by Coomassie blue staining. (I) In B16 and A375 cells with stable VHL knockdown, plasmids expressing the ubiquitin protein (HA‐Ub) and CIP2A (Flag‐CIP2A) were co‐transfected for 48 h. Cells were then treated with 5 µM PF for 24 h, followed by MG132 for 6 h. After immunoprecipitation using an anti‐Flag antibody, and the ubiquitination of CIP2A protein was detected using an anti‐HA antibody.

As a key regulatory factor in the UPS, E3 ligases play a crucial role in selectively recognizing and tagging target proteins.^32^ Therefore, identifying the E3 ligase involved in CIP2A ubiquitination and degradation could reveal the mechanism by which PF induces CIP2A degradation. We first screened for proteins that might interact with CIP2A using the BioGRID database (https://thebiogrid.org/). The potent PF‐interacting protein candidates are shown in Table S2. Among them, six E3 ligases were highly expressed in melanoma cells: BRCA1, CUL3, RNF4, UBR5, CHIP, and von Hippel‒Lindau (VHL). We then established A375 cell lines with stable knockdown of each of these E3 ligases (Figure 6A) and treated them with varying concentrations of PF. The data showed that in melanoma cells with stable knockdown of BRCA1, CUL3, RNF4, UBR5, and CHIP, the reduction in CIP2A levels upon PF treatment was not reversed (Figures 6B and S6). However, knockdown of VHL rescued PF‐induced CIP2A downregulation (Figure 6B,C), indicating that PF likely mediates CIP2A degradation through VHL. Knockdown of VHL in melanoma cells led to increased CIP2A protein expression (Figure 6C), suggesting that VHL may be the natural E3 ligase for CIP2A and that PF might enhance the interaction between VHL and CIP2A, thereby increasing CIP2A instability and degradation. To confirm this hypothesis, we conducted a series of further experiments. Overexpression of VHL resulted in a decrease in CIP2A protein levels (Figure 6D). Immunoprecipitation and immunoblot assays analyses revealed a strong interaction between CIP2A and VHL (Figure 6E). After PF treatment, the degradation of CIP2A increased significantly, along with a notable enhancement in the binding between CIP2A and VHL (Figure 6E), demonstrating that VHL is the E3 ligase for CIP2A and that PF significantly enhances the interaction between CIP2A and VHL. Streptavidin–biotin pull‐down and immunoblot assays analyses further showed that PF‐Biotin could simultaneously capture both CIP2A and VHL (Figure 6F), indicating the formation of a PF‒CIP2A‒VHL ternary complex.

To explore the mechanism of PF‒CIP2A‒VHL complex formation, we conducted further studies. Previous research has shown that VHL forms a ternary complex with transcription elongation factor C (TCEB1) and transcription elongation factor B (TCEB2), known as VCB.^41^ VCB possesses ubiquitin ligase activity and targets proteins for proteasomal degradation. We recombinantly expressed and purified CIP2A (amino acids 1−560) along with His‐tagged VHL, TCEB1, and TCEB2 proteins. Pull‐down assays after incubating these recombinant proteins with PF‐Biotin in vitro demonstrated that PF‐Biotin directly interacts with CIP2A, while VHL, TCEB1, and TCEB2 in the VCB complex do not bind to PF (Figure 6G). His pull‐down assays revealed that CIP2A interacts with His‐VHL, and this interaction is enhanced with increasing PF concentrations (Figure 6H). These results indicate that PF directly interacts with CIP2A in vitro and enhances the interaction between CIP2A and VHL, forming the PF‒CIP2A‒VHL ternary complex. PF does not directly bind to the VCB complex. These may be the mechanism by which PF promotes CIP2A ubiquitination and degradation.

Considering the central role of polyubiquitination in protein degradation, we co‐transfected B16 and A375 cells with plasmids expressing ubiquitin (HA‐Ub) and CIP2A (Flag‐CIP2A) in melanoma cells. Immunoprecipitation using anti‐Flag antibody followed by detection with anti‐HA antibody showed that knockdown of VHL significantly reduced PF‐induced CIP2A polyubiquitination (Figure 6I), indicating that PF probably induces CIP2A polyubiquitination through VHL.

## 3 DISCUSSION

Existing treatments have shown limited efficacy against MBM. One significant factor restricting the effective treatment of brain metastases is the BBB, which impedes drug delivery to the brain.^3^ Therefore, developing novel therapeutic agents capable of crossing the BBB is crucial for enhancing the prognosis of these patients. In this study, through a drug repositioning strategy and screening of various antipsychotics capable of penetrating the BBB, we identified PF as a potential drug of treating melanoma growth and metastasis, including brain metastases, by targeting CIP2A.

PF has demonstrated anticancer activities in various malignancies, including esophageal squamous cell carcinoma,^14^ non‐small cell lung cancer,^15^ and pancreatic cancer.^42^ However, these studies did not clearly identify its direct target proteins, thus limiting the clinical application of PF as an anticancer agent. A pivotal finding of this study was the identification of CIP2A as a direct target of PF, laying the foundation for a deeper understanding of the antitumor mechanisms of PF. CIP2A is overexpressed in various tumor types,^24^ and we demonstrated that it is also highly expressed in melanoma and is closely associated with metastasis and poor prognosis, highlighting the possibility of developing anticancer drugs by targeting CIP2A.

CIP2A stabilizes c‐Myc by inhibiting PP2A activity, thereby activating a series of pathways that promote cancer progression.^25^ Our previous research showed that PP2A dephosphorylates XPO5 and regulates its transport capacity, thereby promoting the biosynthesis of tumor‐suppressing miRNAs such as miR‐122 and miR‐200b.^12^, ^43^ XPO5 is a shuttle protein that mediates the export of precursor miRNA (pre‐miRNA) from the nucleus to the cytoplasm, a crucial step in miRNA maturation.^44^ In this study, we found that PF alleviates the inhibition of the PP2A catalytic subunit (PP2Ac) by CIP2A. PP2Ac is capable of dephosphorylating XPO5 at Ser416, regulating XPO5's ability to transport pre‐miRNA and promoting the expression of tumor‐suppressing miRNAs.^44^ This might represent another critical mechanism by which PF inhibits melanoma growth and metastasis.

Understanding the molecular mechanisms by which PF downregulates CIP2A protein levels is crucial for elucidating PF's anticancer effects and for developing new therapeutics targeting CIP2A. We found that PF facilitates the degradation of CIP2A via the UPS, which is essential for eliminating aberrant proteins, such as misfolded, inactive, or excessively expressed proteins, thereby maintaining protein homeostasis.^45^ In eukaryotic cells, the primary degradation pathways for damaged proteins or organelles include the lysosomal pathway and the UPS pathway.^45^ Typically, the UPS eliminates short‐lived proteins and soluble misfolded proteins, whereas lysosomes degrade long‐lived proteins, insoluble protein aggregates, entire organelles, and even intracellular parasites through phagocytosis, pinocytosis, or autophagy.^31^, ^45^, ^46^

CIP2A protein can be degraded through both the UPS and the lysosomal pathway in cancer. For example, metformin induces CIP2A degradation via the UPS pathway, thereby affecting tumor metabolic plasticity.^35^ Celastrol directly binds to CIP2A and promotes its interaction with the E3 ligase CHIP, enhancing CIP2A degradation.^40^ The mitochondrial complex I inhibitor IACS‐010759 induces ROS‐dependent dissociation of CIP2A from PP2A, leading to lysosomal degradation of CIP2A.^39^ These studies illustrate the complexity of mechanisms by which different drugs degrade CIP2A. Our study discovered that PF promotes CIP2A degradation via the UPS and preliminarily explored the underlying mechanisms.

The UPS marks target proteins with ubiquitin, which are then recognized and degraded by the proteasome.^30^ E3 ubiquitin ligases determine substrate specificity by recognizing specific target proteins and recruiting E2 and E1 enzymes to transfer ubiquitin molecules to these proteins.^31^, ^32^ VHL is involved in the degradation of various oncogenic proteins, making it a promising target in anticancer drug development.^47^ We reported here that VHL is the E3 ligase for CIP2A in melanoma. Earlier biochemical studies have shown that it forms a ternary complex (VCB) with transcription elongation factor C (TCEB1) and transcription elongation factor B (TCEB2),^48^ which is crucial for VHL's function, possessing ubiquitin ligase activity.^48^, ^49^, ^50^ We found that PF significantly enhances the binding affinity between CIP2A and VHL, forming a PF‒CIP2A‒VHL ternary complex. We also preliminarily elucidated the structural mechanism of PF‒CIP2A‒VHL complex formation. PF can directly interact with CIP2A in vitro and enhance the interaction between VHL and CIP2A, thereby inducing polyubiquitination and degradation of CIP2A. However, it does not bind to the VCB complex. Therefore, this study elucidates the detailed mechanism of PF‐mediated CIP2A degradation, providing a foundational basis for developing drugs targeting CIP2A.

The application of protein degraders to downregulate oncogenic proteins, including those previously considered “undruggable” targets, offers significant advantages by inducing target protein degradation through transient binding rather than competitive inhibition. After promoting the polyubiquitination of the target protein, the degrader dissociates.^51^ Consequently, a single degrader molecule can catalytically degrade numerous pathogenic proteins, achieving high efficacy at low doses.^51^ Proteolysis‐targeting chimera (PROTAC) exemplifies this strategy by utilizing E3 ubiquitin ligases for precise degradation of oncogenic proteins.^52^ In contrast to the larger PROTACs, molecular glues are small molecules that induce or stabilize protein‒protein interactions, facilitating the interaction between E3 ubiquitin ligases and target proteins, leading to ubiquitination and degradation.^53^, ^54^ Molecular glues are monovalent molecules without linkers, mediating protein dimerization through the formation of a ternary complex (target‐molecular glue‐E3 ligase).^53^, ^54^ This mechanism results in non‐saturable kinetics without a hook effect and enhanced bioavailability due to their smaller molecular size.^54^ Our results identified that PF can directly bind to CIP2A, promoting the interaction between CIP2A and VHL, thereby facilitating CIP2A degradation. However, the precise binding mode of PF to CIP2A and the mechanism by which it enhances the CIP2A‒VHL interaction remain to be elucidated. Combination therapy is a pivotal strategy in melanoma therapy, offering benefits such as improved therapeutic efficacy and reduced resistance.^55^ The combination of BRAF inhibitors and MEK inhibitors has significantly enhanced survival in patients with BRAF‐mutant melanoma and has shown some efficacy in treating MBM.^56^ In immunotherapy, the combination of anti‐CTLA‐4 antibody and anti‐PD‐1 antibodies has been approved for the treatment of advanced melanoma, yielding favorable outcomes.^57^, ^58^ Nonetheless, these combination therapies still face limitations such as low response rates and treatment resistance. The activation of the PI3K/AKT pathway and c‐Myc is implicated in therapy resistance. Inhibiting these pathways has been shown to reverse treatment resistance.^59^ CIP2A inhibition can restore PP2A activity, thereby suppressing resistance‐related pathways such as PI3K/AKT and c‐Myc.^60^ Additionally, PF can induce cancer cell death, potentially enhancing antitumor immune responses through increased antigen release. This study demonstrated that PF targets and degrades CIP2A protein, inhibits the PI3K/AKT pathway and c‐Myc activity, and induces tumor cell apoptosis. Thus, combining PF with existing standard treatments might produce synergistic effects in treating melanoma and its metastases.

Relative to lung metastasis in cancer, there is currently less basic research on brain metastasis, which limits effective treatment for MBM patients. Several factors contribute to these limitations. The unique nature of the brain microenvironment and its interactions with metastatic cancer cells make results from simple in vitro models of brain metastasis clinically irrelevant and unlikely to reveal promising therapeutic targets and drugs. Additionally, the study of brain metastasis lacks suitable in vivo models.

Reported in vivo brain metastasis models include spontaneous metastasis models and experimental metastasis models in mice.^61^ Experimental metastatic models include left cardiac injection models, stereotactic intracerebral injection models, and intracarotid artery injection models.^61^ Each modeling method has its advantages and disadvantages. Spontaneous brain metastasis from a primary tumor is ideal as it allows for the study of all steps of the metastatic cascade and is simple to establish. However, the efficiency of this model is very low and unstable. In most cases, mice die from metastasis in other organs before detectable brain metastasis forms. The stereotactic intracerebral injection model's advantage is that it provides reproducible and consistent brain lesion formation, but this method ignores all stages of the metastasis cascade except the secondary outgrowth in the brain. The left cardiac injection model and intracarotid artery injection model establish brain metastasis by injecting tumor cells into the circulation, both recapitulating the second half of the metastatic cascade. The efficiency of forming brain metastasis can be improved through multiple rounds of selection in the brain. The advantages of intracarotid arterial injection include high efficiency in forming brain metastasis, as well as reproducibility and consistency. However, they bypass the first half of the metastatic cascade, including intravasation. Other major drawbacks of the left cardiac injection model include high variability and the potential for bone and lung metastases, causing mice to succumb to lung metastasis before brain metastasis develops. Other drawbacks of intracarotid arterial injection include technical challenges and significant trauma to the mice, with a mortality rate reaching up to 30% when using certain cells to establish the model. We have mastered the surgical technique of establishing a brain metastasis model via intracarotid artery injection. By selecting appropriate modeling methods, we can better study the mechanisms of melanoma cell survival in the circulation and growth and adaptation in the specific brain microenvironment, bringing new therapeutic options and hope to cancer patients with brain metastasis.

Despite the promising results, this study has several limitations that require further research. First, melanoma is characterized by numerous mutations, such as BRAF mutations, but the relationship between PF's activity and these mutations was not explored in this study. Second, the structural basis for CIP2A degradation induced by PF is still unclear. The current data do not clarify whether PF promotes CIP2A degradation through a molecular glue mechanism or allosteric modulation, indicating the need for further structural biology studies. Third, the animal models used in this study have some shortcomings. We used subcutaneous tumor models and experimental metastasis models, but for primary tumors, patient‐derived xenograft models and genetically engineered mice that develop melanoma spontaneously would be more suitable. Additionally, the experimental metastasis models employed are brain/lung tumor‐seeding models, which do not fully capture the entire metastatic cascade. Furthermore, treatment was initiated 7 days after model establishment, meaning the results do not reflect PF's effects on the early stages of metastasis. The role and mechanisms of CIP2A in the development and metastasis of melanoma, as well as its involvement in the mechanisms by which PF inhibits melanoma metastasis, were not clearly articulated in this study. Further experiments, including in vivo animal studies, are essential to clarify these aspects in the future.

In conclusion, our study provides compelling evidence that PF can be repositioned as an effective therapeutic agent for melanoma and its brain metastasis by promoting CIP2A degradation and restoring PP2A activity. These findings lay the experimental groundwork for further research and development of PF as a promising treatment for melanoma, particularly for patients with brain metastasis. Additionally, the high expression of CIP2A is associated with melanoma metastasis and poor prognosis, suggesting that CIP2A inhibitors or degraders could become novel antitumor drugs.

## 4 MATERIALS AND METHODS

### 4.1 Cell cultures

Melanoma cell lines B16, A375, C32, A875, HT144, WM115, and A2058 were cultured in Dulbecco's modified Eagle medium supplemented with 10% fetal bovine serum (PAN‐Biotech, #ST30‐3302) plus 100 U/mL of penicillin and streptomycin at 37°C in an incubator with 5% CO_2_. All cell lines were verified by STR profiling. Luciferase‐expressing brain seeking B16 cell line B16Br‐luc was obtained via the following procedure. A total of 5 × 10^4^ B16‐luc cells were injected into the right intracarotid artery of 8−10 weeks C57BL/6 mice (HFK Bioscience), respectively. Bioluminescence signaling in the brain was measured by non‐invasive In Vivo Imaging System (IVIS, PerkinElmer) after administration of 150 mg/kg D‐luciferin potassium salt via i.p. injection. After visualization of the brain metastasis, the whole brain was collected and minced on poly‐lysine coated 10‐cm cell culture dish. The tumor cells will grow and expand in the dish and they are collected as B16Br‐luc cells.

### 4.2 Subcutaneous tumor models

After melanoma cells were resuspended in HBSS (Hanks' balanced salt solution) and mixed with an equal volume of Matrigel, 2 × 10^5^ B16 cells were subcutaneously inoculated into the right flanks of 6−8 week‐old C57BL/6 mice, and 5 × 10^6^ A375 cells were subcutaneously inoculated into the right flanks of 6−8 week‐old BALB/c nude mice. When the tumor volume reached approximately 100 mm^3^, the mice were randomly divided into three groups, with six mice per group, and treated twice a week with oral administration of PF (15 mg/kg), intraperitoneal injection of TMZ (40 mg/kg), or vehicle. The treatment regimen of PF and TMZ was modified based on previous reports.^14^, ^16^, ^62^ PF was diluted in water/PEG300/ethanol/2% acetic acid in 8:3:3:1 v/v, while TMZ was dissolved in normal saline. Tumor volume was calculated using the formula: tumor volume = 0.5 × width^2^ × length. Tumor volume and body weight of mice were recorded every 3 days. At the end of the treatment period, mice were euthanized under sodium pentobarbital anesthesia. The excised tumor tissues were photographed and fixed in 4% paraformaldehyde for subsequent immunohistochemistry.

### 4.3 Establishment of experimental metastasis models

For the brain metastasis model, 1 × 10^4^ brain‐seeking B16Br‐luc cells were resuspended in 100 µL of HBSS and injected into the right intracarotid artery of female C57BL/6 mice. For the lung metastasis model, 1 × 10^5^ B16‐luc cells were injected into the tail vein of C57BL/6 mice. Seven days post‐inoculation, the mice were randomly divided into three groups of six and treated according to the same regimen as in the subcutaneous tumor treatment experiment. At specified time points, bioluminescence images of metastasis were captured using an Aniview 100 (Biolight Biotechnology Co., Ltd.) 10 min after intraperitoneal injection of D‐luciferin potassium salt (150 mg/kg). At the end of the experiment, the brains and lungs were harvested and fixed in 4% formalin for H&E staining.

### 4.4 Toxicity evaluation

Preliminary toxicity testing experiments were conducted using healthy C57BL/6 mice without tumor modeling. The mice were orally gavaged with 15 mg/kg of PF twice a week. Four hours after the last administration, the mice were euthanized and the hearts, livers, spleens, lungs, and kidneys of the mice were fixed in 4% paraformaldehyde and subjected to H&E staining.

### 4.5 Bioinformatics analysis methods

TCGA database (https://cancergenome.nih.gov/) was used to evaluate the expression levels of CIP2A and analyze the clinical characteristics and prognosis of melanoma patients. Additionally, expression levels and correlation analyses for CIP2A in melanoma were retrieved from BEST (https://rookieutopia.hiplot.com.cn/app direct/BEST/).^63^ Kaplan‒Meier survival analysis was conducted to assess the relationship between the expression of CIP2A and patient prognosis. This study utilizes publicly accessible data, which can be found on GEO repository (http://www.ncbi.nlm.nih.gov/geo/) under the following accession numbers: GSE111766, GSE44660, GSE15605, GSE98394, GSE133713, and GSE22153.

### 4.6 Statistical analysis

Data are presented as mean ± standard deviation or standard error of the mean. Data analysis and visualization were performed using GraphPad Prism 5 software (version 8.0.1, GraphPad Software). Statistical significance between two groups was assessed using a two‐tailed Student's *t*‐test. For comparisons among multiple groups, one‐way analysis of variance followed by Dunnett's test was used. Survival significance was displayed using Kaplan‒Meier curves and results were calculated based on the log‐rank test. A *p*‐value < 0.05 was considered statistically significant. Statistical significance is indicated as ^*^ *p* < 0.05, ^**^ *p* < 0.01, ^***^ *p* < 0.001.

## AUTHOR CONTRIBUTIONS

*Conceptualization*: Yong Peng, Yong Xia, and Fuyan Xu *Methodology*: Fuyan Xu, Zhu Yuan, Jiao Li, Tingting Zhang, Zhang Li, Yang Yang, and Wenchen Pu *Investigation*: Fuyan Xu, Jiao Li, Min Ai, Tingting Zhang, Yue Ming, Yang Yang, and Xiaomin Xu *Funding acquisition*: Yong Peng and Yong Xia *Project administration*: Yong Peng and Zhu Yuan *Supervision*: Yong Peng, Yong Xia, Zhang Li, Yucheng Qi, and Zhang Li *Writing*: Fuyan Xu, Jiao Li, Yong Xia, and Yong Peng. All the authors have read and approved the manuscript.

## CONFLICT OF INTEREST STATEMENT

Author Yong Peng is an Editorial board member of MedComm. Author Yong Peng was not involved in the journal's review of or decisions related to this manuscript. The remaining authors declare they have no conflicts of interest.

## ETHICS STATEMENT

All mouse experiments conducted in this study were approved by the Institutional Animal Care and Use Committee of West China Hospital, Sichuan University (no. 20211380A).

## Supporting information



Supporting Information

## Data Availability

All data included in this study are available from the corresponding author upon a reasonable request.
