# Supplementary material for: Penfluridol inhibits melanoma growth and metastasis through enhancing von Hippel‒Lindau tumor suppressor‐mediated cancerous inhibitor of protein phosphatase 2A (CIP2A) degradation
Source: MedComm (2020). 2024 Oct 13;5(10):e758. doi: 10.1002/mco2.758 (PMC11470999; doi:10.1002/mco2.758)
Supplement: Supplementary file 1 — Supporting Information [file MCO2-5-e758-s001.docx]

**Supplementary Materials**

**Penfluridol inhibits melanoma growth and metastasis through enhancing Von Hippel-Lindau tumor suppressor (VHL)-mediated Cancerous inhibitor of protein phosphatase 2A (CIP2A) degradation**

Fuyan Xu^1#^, Jiao Li^1#^, Min Ai^1^, Tingting Zhang^1^, Yue Ming^1^, Cong Li^2^, Wenchen Pu^1^, Yang Yang^1^, Zhang Li^1^, Yucheng Qi^1^, Xiaomin Xu^1^, Qingxiang Sun^2^, Zhu Yuan^2^*, Yong Xia^3^*, Yong Peng^1,4^*

^1^Laboratory of Molecular Oncology, Frontiers Science Center for Disease-Related Molecular Network, State Key Laboratory of Biotherapy, West China Hospital, Sichuan University, Chengdu, 610041

^2^Department of Biotherapy, Cancer Center and State Key Laboratory of Biotherapy, West China Hospital, Sichuan University, Chengdu 610041

^3^Rehabilitation Medicine Center, State Key Laboratory of Biotherapy, West China Hospital, Sichuan University, Chengdu 610041

^4^Frontier Medical Center, Tianfu Jincheng Laboratory, Chengdu, 610212, China

^#^ Fuyan Xu and Jiao Li contributed equally to this work.

***Correspondence:**

**Yong Peng,** 387 Hemin Street, High-tech Zone, Chengdu, Sichuan, 610213, China, Email: yongpeng@scu.edu.cn;

**Yong Xia**, No.17 People's South Road, Chengdu, Sichuan,610041, China, Email: yxia4@scu.edu.cn;

**Zhu Yuan**, No.17 People's South Road, Chengdu, Sichuan, 610041, China, Email: yuanzhu@scu.edu.cn

**Supporting Information Text**

**Reagents**

Penfluridol (#S4151), Chlorpromazine (#S2456), Fluphenazine (#S4569), Trifluoperazine (#S3201), Fluoxetine (#S1333), Sertraline (#S4053), Pimozide (#S4358), Haloperidol (#S1920), Chlorprothixene (#S5399), Thioridazine (#S5563), Fluspirilene (#E1410), and Perphenazine (#S4731) were from purchased Selleck; Zuclopenthixol (#Z303928) and Pimavanserin (#P177243) from Aladdin; Temozolomide (#HY-17364), MG132 (#HY-12359), and Chloroquine (#HY-17589A) from MCE; Cycloheximide (#5087390001) from Sigma-Aldrich.

**Plasmid construction and cell transfection**

To construct knockdown plasmids, the annealed oligos targeting coding region were designed and inserted into pLKO.1 TRC cloning plasmid (Addgene). The full-length coding sequence of human and mouse CIP2A and VHL genes were cloned into pCDH-CMV-MCS-EF1-Flag (System Biosciences) vector which used Phanta Super-fidelity DNA polymerase (Vazyme, #P501-d1). To construct plasmids for the overexpression of CIP2A after knockdown, site-directed mutagenesis primers were designed according to the instruction manual of Agilent (Catalog, #200518). PCR reactions were performed using site-directed mutagenesis primers and overexpression plasmids as templates. For each reaction, 1 μL *Dpn*I (NEB, #R0176V) was added and incubated at 37°C overnight for digestion. The primers were shown in Table S3. The cells were transfected with plasmids using Lipofectamine 2000 (Invitrogen, #11668019) according to the manufacturer’s instructions.

**RT-qPCR**

Total RNA was extracted using RNAiso Plus reagent (TaKaRa, #9108) according to the manufacturer’s instructions, and 1 μg of RNA was reversely transcribed using PrimeScriptTM RT reagent kit (TaKaRa, #RR047A). RT-qPCR was performed in triplicates with indicated primers using TB Green® Premix Ex Taq^TM^ (TaKaRa, #RR820A). The primers are listed in Table S3.

**Cell viability, colony formation and apoptosis assays**

Cell viabilities were evaluated using MTT assay. In the colony formation assay, cells were plated in 6-well plates and allowed to adhere overnight. After incubation with PF (2 μM) for 10 days, the cells were visualized after crystal violet (0.1%) staining and clone numbers in each group were counted. Cell apoptosis was determined by the Annexin V-PE/7-AAD Apoptosis Detection Kit. In brief, cells suspended in PBS were stained with Annexin V-PE and 7-AAD for 15 min in the dark and the apoptosis was analyzed on a BD Accuri^TM^ C6 flow cytometer.

**Cell migration/invasion assays**

For cell migration and invasion assays, Transwell chamber (Millipore, #FAL-353097) with or without diluted Matrigel (Corning, #356231). Matrigel was diluted in PBS at a ratio of 1:8. Cells were resuspended in DMEM containing 5% BSA. Then, 200 μL of the cell suspension with varying concentrations of the drug was added to the upper chamber, and 800 μL of medium containing 10% FBS and the drug was added to the lower chamber. After 24 h, cells that had passed through the chamber membrane were fixed with 4% paraformaldehyde for 30 min and stained with 0.5% crystal violet solution for 30 min. Finally, the number of cells that passed through the membrane was photographed and counted under a microscope.

**Streptavidin pull-down assays and high-resolution mass spectrum ananlysis.**

Cells were suspended in lysis buffer and homogenized. The supernatant was collected and incubated with 1 μM PF-Biotin or 1 μM PEG3-biotin for 12 h. Subsequently, the mixture was incubated with streptavidin magnetic beads (Thermo, #65601) at 4°C for 2 h, followed by washing three times with wash buffer. Proteins bound to the beads were harvested and separated by SDS-PAGE and stained with Coomassie Brilliant Blue. Differential protein bands of interest were excised and subjected to further processing as described before.^65^ Finally, peptide samples were subjected to high-resolution mass spectrometry (Thermo, Exploris^TM^ 480) for qualitative analysis.

**Immunoblotting**

Cells were lysed using RIPA lysis buffer containing protease and phosphatase inhibitors (Beyotime Biotechnology, #P0013B) and sonicated. After centrifugation at 13,000 rpm for 15 minutes at 4 °C, the concentration of protein in the supernatant was quantified using the Pierce™ BCA Protein Assay Kit (Beyotime Biotechnology, #P0012). Equal amounts of protein were separated on 6%-15% SDS-PAGE gels and transferred onto PVDF membranes (Millipore). The membranes were incubated with primary antibodies overnight at 4°C, followed by incubation with HRP-conjugated anti-mouse or anti-rabbit secondary antibodies at room temperature for 1 h. The proteins were detected using a ChemiDoc imaging system (Bio-Rad). The sources of the antibodies are shown in Table S4.

**Cellular thermal shift assay (CETSA)**

After treating B16 and A375 cells with 10 μM PF or DMSO for 4 h, cells from different groups were evenly distributed into 8 PCR tubes and heated at 40°C, 42°C, 44°C, 46°C, 48°C, 50°C, and 52°C for 3 minutes, followed by incubation at room temperature for 3 min. Subsequently, the cells were repeatedly frozen and thawed using liquid nitrogen and a 37°C water bath for 5 cycles to obtain cell lysates. The lysates were then centrifuged at 20,000g for 20 min at 4°C to collect the supernatant. immunoblot assays were performed to detect non-denatured CIP2A protein in different groups.

**Drug affinity responsive target stability (DARTS)**

A375 cells were lysed on ice to obtain cell lysates, which were subsequently divided into two groups: one treated with DMSO and the other with 10 μM PF. After incubation on a rotator at room temperature for 30 min, different concentrations of proteinase K were added and incubated for 5 min. Digestion was terminated by adding 5 × SDS loading buffer and heating at 95°C for 10 min. The samples were then subjected to SDS-PAGE, followed by immunoblot assays analysis to detect CIP2A.

**Protein expression and purification**

After cloning the cDNA encoding CIP2A amino acids 1-560 into the pGEX-6P-1 vector, it was transformed into *E. coli* BL21(DE3). The *E. coli* cells were cultured in LB medium until reaching an OD600 of 0.6~0.8, followed by induction with 0.1 mM isopropyl β-D-1-thiogalactopyranoside at 16°C for 16 h. Subsequently, the cells were centrifuged for 15 min, resuspended in lysis buffer, and the proteins were purified using glutathione-agarose beads. The GST tag was then cleaved with TEV protease. The purified proteins underwent further size-exclusion chromatography using a Superdex 200 Increase 10/300 GL column (GE Healthcare).

**Isothermal titration calorimetry (ITC)**

The MicroCal ITC200 calorimeter was employed to assess the interaction between PF and CIP2A. PF (1 mM, dissolved in 20 mM Tris-HCl, pH 7.5, 10 mM NaCl, 2% DMSO, 4% Tween 20) was titrated into 50 μM recombinant hu-CIP2A (1-560). The data were analyzed using the one-site binding model in Origin 7.

**Immunoprecipitation**

Resuspend the cells in lysis buffer and lyse them on ice for 20 min, followed by homogenization. Centrifuge the lysate, then incubate the supernatant with the indicated antibodies or corresponding IgG overnight at 4°C. Add protein A/G Sepharose beads and incubate at 4°C for 3 h. Wash the beads three times and resuspend them in loading buffer and boil at 95°C for 10 min. Detect the corresponding proteins by immunoblot assays.

**Ubiquitination assay**

After transfecting melanoma cells with HA-tagged ubiquitin plasmid and Flag-tagged CIP2A plasmid for 48 h, treat the cells with 5 μM PF for 24 h, followed by 20 μM MG132 for 6 h. Collect the cells and lyse them completely in IP lysis buffer using sonication. Centrifuge at 13,000 rpm for 15 min at 4°C, then incubate the supernatant with pre-blocked anti-FLAG M2 affinity gel (Sigma) at 4°C for 6 h. Wash the gel three times with wash buffer, resuspend it in loading buffer, and boil at 95°C for 10 min. Finally, use anti-HA and anti-Flag antibodies to detect the expression of the corresponding proteins by immunoblot assays.

**Synthesis of Biotin-Labeled Penfluridol probe**

Design and chemical synthesis route of PF-Biotin (1b) is shown in Figure S7. The synthesis of PF-propynyl (PF-propynyl, 1a) as the first intermediate in the PF-Biotin synthetic pathway proceeds as follows: Place sodium hydride in a dry reaction flask under a nitrogen atmosphere and place it in an ice bath, Add DMF and stir for 2 min, Add PF dissolved in DMF, remove the ice bath, and stir at room temperature for 30 min, Add propynyl bromide dissolved in DMF and react at room temperature for 4 h until a small polar product spot forms, Add 20 mL of water and extract with ethyl acetate. Wash the organic phase with saturated sodium chloride, dry over anhydrous sodium sulfate, and concentrate, Purify by column chromatography using a gradient (DCM: MeOH = 100:1 to 50:1) to obtain PF-propynyl 1a, Characterize the product using ^1^H-NMR, ^13^C-NMR, and mass spectrometry to confirm the correct structure of PF-propynyl 1a. Dissolve compound 1a in a 1 mL THF/water (1:1) solution, Add copper sulfate, sodium ascorbate, and N3-PEG3-Biotin, and react at room temperature for 3 h, as shown in the following schematic, Evaporate to dryness, dissolve the residue in a DCM/MeOH mixture, and centrifuge. Collect the supernatant. Separate the product by preparative thin-layer chromatography (DCM: MeOH = 10%:15% triethylamine). Purify the product by HPLC, and characterize it using ^1^H-NMR, ^13^C-NMR, and mass spectrometry. Confirm the structure and size are correct, naming it PF-Biotin (1b).

**Table S1: The proteins interacting with PF identified by mass spectrometry**

| Accession | Gene Name | Coverage [%] | Unique Peptides | MW [kDa] | Score |
| --- | --- | --- | --- | --- | --- |
| P55060 | CIP2A | 6 | 5 | 110.3 | 216.36 |
| O14980 | XPO1 | 3 | 3 | 123.3 | 113.38 |
| Q92616 | GCN1 | 2 | 5 | 292.6 | 111.39 |
| O00410 | IPO5 | 3 | 3 | 123.6 | 66.87 |
| O43156 | TTI1 | 2 | 2 | 122 | 65.61 |
| Q08554 | DSC1 | 2 | 1 | 99.9 | 65.21 |
| P20930 | FLG | 1 | 1 | 434.9 | 33.3 |
| Q6P9B9 | INTS5 | 1 | 1 | 107.9 | 25.84 |
| Q86YZ3 | HRNR | 1 | 1 | 282.2 | 23.76 |
| O14983 | ATP2A1 | 1 | 1 | 110.2 | 12.46 |
| P16615 | ATP2A2 | 1 | 1 | 114.7 | 8.46 |
| P57740 | NUP107 | 1 | 1 | 106.3 | 2.31 |
| Q9UIA9 | XPO7 | 1 | 1 | 123.8 | 2.12 |
| P78527 | PRKDC | 0 | 1 | 468.8 | 1.92 |

**Table S2: Potential interacting proteins of CIP2A obtained from BioGRID**

| **Interactor** | **Organism** | **Description** |
| --- | --- | --- |
| NSP14 | SARS-CoV-2 | Guanine-N7 methyltransferase; Non-structural protein 14; 3-to-  5 exonuclease |
| NSP15 | SARS-CoV-2 | Uridylate-specific endoribonuclease; Non-structural protein 15; endoRNAse |
| NSP3 | SARS-CoV-2 | Non-structural protein 3 |
| NSP6 | SARS-CoV-2 | Non-structural protein 6 |
| ORF10 | SARS-CoV-2 | ORF10 protein |
| RAB18 | M. musculus | RAB18, member RAS oncogene family |
| RNF183 | M. musculus | Ring finger protein 183 |
| ORF4 | HHV-8 | Complement control protein |
| KIAA1524 | H. sapiens | KIAA1524 |
| ALDH3A2 | H. sapiens | Aldehyde dehydrogenase 3 family, member A2 |
| PPP2R5C | H. sapiens | Protein phosphatase 2, regulatory subunit B', gamma |
| CA14 | H. sapiens | Carbonic anhydrase XIV |
| CD274 | H. sapiens | CD274 molecule |
| CEACAM21 | H. sapiens | Carcinoembryonic antigen-related cell adhesion molecule 21 |
| COMTD1 | H. sapiens | Catechol-O-methyltransferase domain containing 1 |
| EGFR | H. sapiens | Epidermal growth factor receptor |
| MYC | H. sapiens | V-myc avian myelocytomatosis viral oncogene homolog |
| SCN2B | H. sapiens | Sodium channel, voltage-gated, type II, beta subunit |
| VSIG2 | H. sapiens | V-set and immunoglobulin domain containing 2 |
| AVPR2 | H. sapiens | Arginine vasopressin receptor 2 |
| BRCA1 | H. sapiens | Breast cancer 1, early onset |
| CD83 | H. sapiens | CD83 molecule |
| CHRM4 | H. sapiens | Cholinergic receptor, muscarinic 4 |
| EDNRB | H. sapiens | Endothelin receptor type B |
| EFNA4 | H. sapiens | Ephrin-A4 |
| FZD10 | H. sapiens | Frizzled class receptor 10 |
| HTR2C | H. sapiens | 5-hydroxytryptamine (serotonin) receptor 2C, G protein-coupled |
| IL1R2 | H. sapiens | Nterleukin 1 receptor, type II |
| ITFG3 | H. sapiens | Integrin alpha FG-GAP repeat containing 3 |
| LRFN4 | H. sapiens | Leucine rich repeat and fibronectin type III domain containing 4 |
| LRRC4 | H. sapiens | Leucine rich repeat containing 4 |
| LRRC59 | H. sapiens | Leucine rich repeat containing 59 |
| LRRC61 | H. sapiens | leucine rich repeat containing 61 |
| NPY2R | H. sapiens | Neuropeptide Y receptor Y2 |
| PNKD | H. sapiens | Paroxysmal nonkinesigenic dyskinesia |
| PPP2R1A | H. sapiens | Protein phosphatase 2, regulatory subunit A, alpha |
| PTGER3 | H. sapiens | Prostaglandin E receptor 3 (subtype EP3) |
| SIGLECL1 | H. sapiens | SIGLEC family like 1 |
| SLC30A4 | H. sapiens | Solute carrier family 30 (zinc transporter), member 4 |
| STUB1 | H. sapiens | STIP1 homology and U-box containing protein 1, E3 ubiquitin protein ligase |
| TACSTD2 | H. sapiens | Tumor-associated calcium signal transducer 2 |
| TOPBP1 | H. sapiens | Topoisomerase (DNA) II binding protein 1 |
| VASN | H. sapiens | Vasorin |
| VIPR2 | H. sapiens | Vasoactive intestinal peptide receptor 2 |
| ABCE1 | H. sapiens | ATP-binding cassette, sub-family E (OABP), member 1 |
| ACPP | H. sapiens | Acid phosphatase, prostate |
| ACTR1A | H. sapiens | ARP1 actin-related protein 1 homolog A, centractin alpha (yeast) |
| ARMC5 | H. sapiens | Armadillo repeat containing 5 |
| ASXL1 | H. sapiens | Additional sex combs like transcriptional regulator 1 |
| B3GNT8 | H. sapiens | UDP-GlcNAc:betaGal beta-1,3-N-acetylglucosaminyltransferase 8 |
| BAI1 | H. sapiens | Brain-specific angiogenesis inhibitor 1 |
| BAP1 | H. sapiens | BRCA1 associated protein-1 (ubiquitin carboxy-terminal hydrolase) |
| BCAR1 | H. sapiens | Breast cancer anti-estrogen resistance 1 |
| BTNL9 | H. sapiens | Butyrophilin-like 9 |
| C19ORF38 | H. sapiens | Chromosome 19 open reading frame 38 |
| C3ORF18 | H. sapiens | Chromosome 3 open reading frame 18 |
| C5AR1 | H. sapiens | Complement component 5a receptor 1 |
| CAPZB | H. sapiens | Capping protein (actin filament) muscle Z-line, beta |
| CCAR1 | H. sapiens | Cell division cycle and apoptosis regulator 1 |
| CD40 | H. sapiens | CD40 molecule, TNF receptor superfamily member 5 |
| CDH1 | H. sapiens | Cadherin 1, type 1, E-cadherin (epithelial) |
| CDK1 | H. sapiens | Cyclin-dependent kinase 1 |
| CDK12 | H. sapiens | Cyclin-dependent kinase 12 |
| CENPC | H. sapiens | Centromere protein C |
| CEP104 | H. sapiens | Centrosomal protein 104kDa |
| CEP128 | H. sapiens | Centrosomal protein 128kDa |
| CEP152 | H. sapiens | Centrosomal protein 152kDa |
| CEP192 | H. sapiens | Centrosomal protein 192kDa |
| CFTR | H. sapiens | Cystic fibrosis transmembrane conductance regulator (ATP-binding cassette sub-family C, member 7) |
| CLEC16A | H. sapiens | C-type lectin domain family 16, member A |
| CNTROB | H. sapiens | Centrobin, centrosomal BRCA2 interacting protein |
| CSPP1 | H. sapiens | Centrosome and spindle pole associated protein 1 |
| CSTF2T | H. sapiens | Cleavage stimulation factor, 3' pre-RNA, subunit 2, 64kDa, tau variant |
| CUL3 | H. sapiens | Cullin 3 |
| CXCR4 | H. sapiens | Chemokine (C-X-C motif) receptor 4 |
| CYB5B | H. sapiens | Cytochrome b5 type B (outer mitochondrial membrane) |
| DDRGK1 | H. sapiens | DDRGK domain containing 1 |
| DHFRL1 | H. sapiens | Dihydrofolate reductase-like 1 |
| DHX29 | H. sapiens | DEAH (Asp-Glu-Ala-His) box polypeptide 29 |
| DNAJA1 | H. sapiens | DnaJ (Hsp40) homolog, subfamily A, member 1 |
| DUSP12 | H. sapiens | Dual specificity phosphatase 12 |
| DYNC1LI1 | H. sapiens | Dynein, cytoplasmic 1, light intermediate chain 1 |
| EFNA5 | H. sapiens | Ephrin-A5 |
| EFNB1 | H. sapiens | Ephrin-B1 |
| EGLN3 | H. sapiens | Egl-9 family hypoxia-inducible factor 3 |
| ENOSF1 | H. sapiens | Enolase superfamily member 1 |
| F2RL1 | H. sapiens | Coagulation factor II (thrombin) receptor-like 1 |
| FPR1 | H. sapiens | Formyl peptide receptor 1 |
| FXYD3 | H. sapiens | FXYD domain containing ion transport regulator 3 |
| FXYD6 | H. sapiens | FXYD domain containing ion transport regulator 6 |
| G3BP1 | H. sapiens | GTPase activating protein (SH3 domain) binding protein 1 |
| GAN | H. sapiens | Gigaxonin |
| GBF1 | H. sapiens | Golgi brefeldin A resistant guanine nucleotide exchange factor 1 |
| GCGR | H. sapiens | Glucagon receptor |
| GOLGA4 | H. sapiens | Golgin A4 |
| GPR17 | H. sapiens | G protein-coupled receptor 17 |
| GPR182 | H. sapiens | G protein-coupled receptor 182 |
| GSK3A | H. sapiens | Glycogen synthase kinase 3 alpha |
| GSK3B | H. sapiens | Glycogen synthase kinase 3 beta |
| GYPA | H. sapiens | Glycophorin A (MNS blood group) |
| HCST | H. sapiens | Hematopoietic cell signal transducer |
| HDAC1 | H. sapiens | Histone deacetylase 1 |
| IMPDH2 | H. sapiens | IMP (inosine 5'-monophosphate) dehydrogenase 2 |
| JTB | H. sapiens | Jumping translocation breakpoint |
| KBTBD4 | H. sapiens | Kelch repeat and BTB (POZ) domain containing 4 |
| KDF1 | H. sapiens | Keratinocyte differentiation factor 1 |
| KDM1A | H. sapiens | Lysine (K)-specific demethylase 1A |
| KIR2DL4 | H. sapiens | Killer cell immunoglobulin-like receptor, two domains, long cytoplasmic tail, 4 |
| LAMB2 | H. sapiens | Laminin, beta 2 (laminin S) |
| LAMP1 | H. sapiens | Lysosomal-associated membrane protein 1 |
| LMAN1 | H. sapiens | Lectin, mannose-binding, 1 |
| LMBR1L | H. sapiens | Limb development membrane protein 1-like |
| MED4 | H. sapiens | Mediator complex subunit 4 |
| MFN2 | H. sapiens | Mitofusin 2 |
| MFNG | H. sapiens | O-fucosylpeptide 3-beta-Nacetyl glucosaminyltransferase |
| MLNR | H. sapiens | Motilin receptor |
| NDC80 | H. sapiens | NDC80 kinetochore complex component |
| NEK2 | H. sapiens | NIMA-related kinase 2 |
| NPTN | H. sapiens | Neuroplastin |
| NTRK1 | H. sapiens | Neurotrophic tyrosine kinase, receptor, type 1 |
| NXF1 | H. sapiens | Nuclear RNA export factor 1 |
| OFD1 | H. sapiens | Oral-facial-digital syndrome 1 |
| OPALIN | H. sapiens | Oligodendrocytic myelin paranodal and inner loop protein |
| P2RY10 | H. sapiens | Purinergic receptor P2Y, G-protein coupled, 10 |
| P2RY6 | H. sapiens | Pyrimidinergic receptor P2Y, G-protein coupled, 6 |
| P2RY8 | H. sapiens | Purinergic receptor P2Y, G-protein coupled, 8 |
| PARP1 | H. sapiens | Poly (ADP-ribose) polymerase 1 |
| PHF21A | H. sapiens | PHD finger protein 21A |
| PHLPP1 | H. sapiens | PH domain and leucine rich repeat protein phosphatase 1 |
| PIK3R1 | H. sapiens | Phosphoinositide-3-kinase, regulatory subunit 1 (alpha) |
| PLEKHA4 | H. sapiens | Pleckstrin homology domain containing, family A (phosphoinositide binding specific) member 4 |
| POC1B | H. sapiens | POC1 centriolar protein B |
| PPEF2 | H. sapiens | Protein phosphatase, EF-hand calcium binding domain 2 |
| PPP1R10 | H. sapiens | Protein phosphatase 1, regulatory subunit 10 |
| PRNP | H. sapiens | Prion protein |
| PRRC2A | H. sapiens | Proline-rich coiled-coil 2A |
| PSMA1 | H. sapiens | Proteasome (prosome, macropain) subunit, alpha type, 1 |
| PSMA3 | H. sapiens | Proteasome (prosome, macropain) subunit, alpha type, 3 |
| PTK2 | H. sapiens | Protein tyrosine kinase 2 |
| PTPRM | H. sapiens | Protein tyrosine phosphatase, receptor type, M |
| PTPRN | H. sapiens | Protein tyrosine phosphatase, receptor type, N |
| RAB11A | H. sapiens | RAB11A, member RAS oncogene family |
| RAB7A | H. sapiens | RAB7A, member RAS oncogene family |
| RBM5 | H. sapiens | RNA binding motif protein 5 |
| RCOR1 | H. sapiens | REST corepressor 1 |
| RICTOR | H. sapiens | RPTOR independent companion of MTOR, complex 2 |
| RNF2 | H. sapiens | Ring finger protein 2 |
| RNF4 | H. sapiens | Ring finger protein 4 |
| RPA1 | H. sapiens | Replication protein A1, 70kDa |
| RPA2 | H. sapiens | Replication protein A2, 32kDa |
| RPA3 | H. sapiens | Replication protein A3, 14kDa |
| RPS27A | H. sapiens | Ribosomal protein S27a |
| SEMA4C | H. sapiens | Sema domain, immunoglobulin domain (Ig), transmembrane domain (TM) and short cytoplasmic domain, (semaphorin) 4C |
| SGOL1 | H. sapiens | Shugoshin-like 1 (S. pombe) |
| SLC25A28 | H. sapiens | Solute carrier family 25 (mitochondrial iron transporter), member 28 |
| SMC5 | H. sapiens | Structural maintenance of chromosomes 5 |
| SNCA | H. sapiens | Synuclein, alpha (non A4 component of amyloid precursor) |
| ST6GAL1 | H. sapiens | ST6 beta-galactosamide alpha-2,6-sialyltranferase 1 |
| STIP1 | H. sapiens | Stress-induced phosphoprotein 1 |
| STOM | H. sapiens | Stomatin |
| SYMPK | H. sapiens | Symplekin |
| SYNCRIP | H. sapiens | Synaptotagmin binding, cytoplasmic RNA interacting protein |
| TDRD3 | H. sapiens | Tudor domain containing 3 |
| TIPARP | H. sapiens | TCDD-inducible poly (ADP-ribose) polymerase |
| TNFSF13B | H. sapiens | Tumor necrosis factor (ligand) superfamily, member 13b |
| TNIP2 | H. sapiens | TNFAIP3 interacting protein 2 |
| TNRC6C | H. sapiens | Trinucleotide repeat containing 6C |
| TPST2 | H. sapiens | Tyrosylprotein sulfotransferase 2 |
| TRIM67 | H. sapiens | Tripartite motif containing 67 |
| TSPAN10 | H. sapiens | Tetraspanin 10 |
| TUBB | H. sapiens | Tubulin, beta class I |
| UBR5 | H. sapiens | Ubiquitin protein ligase E3 component n-recognin 5 |
| UCHL1 | H. sapiens | Ubiquitin carboxyl-terminal esterase L1 (ubiquitin thiolesterase) |
| UNC5B | H. sapiens | Unc-5 homolog B (C. elegans) |
| UNC93B1 | H. sapiens | Unc-93 homolog B1 (C. elegans) |
| VCL | H. sapiens | Vinculin |
| VCP | H. sapiens | Valosin containing protein |
| VSIG1 | H. sapiens | V-set and immunoglobulin domain containing 1 |
| WDR36 | H. sapiens | WD repeat domain 36 |
| WWC3 | H. sapiens | WWC family member 3 |
| XPO1 | H. sapiens | Exportin 1 |
| ZC2HC1A | H. sapiens | Zinc finger, C2HC-type containing 1A |
| ZRANB1 | H. sapiens | Zinc finger, RAN-binding domain containing 1 |
| NF1 | H. sapiens | Neurofibromin 1 |
| VHL | H. sapiens | Von Hippel-Lindau tumor suppressor, E3 ubiquitin protein ligase |

**Table S3: List of Primers**

| **Primers for shRNA construction** | **Sequence (5’-3’)** |
| --- | --- |
| Human-sh1-CIP2A | CCGGTGCGGCACTTGGAGGTAATTTCTCGAGAAATTACCTCCAAGTGCCGCATTTTT |
|  | AATTAAAAATGCGGCACTTGGAGGTAATTTCTCGAGAAATTACCTCCAAGTGCCGCA |
| Human-sh2-CIP2A | CCGGCGCAGCAAGTTGAATCAGAAACTCGAGTTTCTGATTCAACTTGCTGCGTTTTT |
|  | AATTAAAAACGCAGCAAGTTGAATCAGAAACTCGAGTTTCTGATTCAACTTGCTGCG |
| Mouse-sh1-CIP2A | CCGGGCCCATATAGATGACTTAATTCTCGAGAATTAAGTCATCTATATGGGCTTTTT |
|  | AATTAAAAAGCCCATATAGATGACTTAATTCTCGAGAATTAAGTCATCTATATGGGC |
| Mouse-sh2-CIP2A | CCGGCCACAGTTTAAGTGGTGGAAACTCGATTTCCACCACTTAAACTGTGGTTTTT |
|  | AATTAAAAACCACAGTTTAAGTGGTGGAAACTCGAGTTTCCACCACTTAAACTGTGG |
| Human-sh1-VHL | CCGGTATCACACTGCCAGTGTATACCTCGAGGTATACACTGGCAGTGTGATATTTTT |
|  | AATTAAAAATATCACACTGCCAGTGTATACCTCGAGGTATACACTGGCAGTGTGATA |
| Mouse-sh1-VHL | CCGGGACATCGTCAGGTCACTCTATCTCGAGATAGAGTGACCTGACGATGTC TTTTT |
|  | AATTAAAAAGACATCGTCAGGTCACTCTATCTCGAGATAGAGTGACCTGACGATGTC |
| Human-sh1-UBR5 | CCGGGCAGGATTGTAGGTTACTTAGCTCGAGCTAAGTAACCTACAATCCTGCTTTTT |
|  | AATTAAAAAGCAGGATTGTAGGTTACTTAGCTCGAGCTAAGTAACCTACAATCCTGC |
| Human-sh1-BRCA1 | CCGGGAGTATGCAAACAGCTATAATCTCGAGATTATAGCTGTTTGCATACTCTTTTT |
|  | AATTAAAAAGAGTATGCAAACAGCTATAATCTCGAGATTATAGCTGTTTGCATACTC |
| Human-sh1-RNF4 | CCGGGACAAGCTCAGAAGCGAACTCCTCGAGGAGTTCGCTTCTGAGCTTGTCTTTTT |
|  | AATTAAAAAGACAAGCTCAGAAGCGAACTCCTCGAGGAGTTCGCTTCTGAGCTTGTC |
| Human-sh1-CUL3 | CCGGGACTATATCCAGGGCTTATTGCTCGAGCAATAAGCCCTGGATATAGTCTTTTT |
|  | AATTAAAAAGACTATATCCAGGGCTTATTGCTCGAGCAATAAGCCCTGGATATAGTC |
| Human-sh1-CHIP | CCGGGCAGTCTGTGAAGGCGCACTTCTCGAGAAGTGCGCCTTCACAGACTGCTTTTT |
|  | AATTAAAAAGCAGTCTGTGAAGGCGCACTTCTCGAGAAGTGCGCCTTCACAGACTGC |
| **Primers for qPCR** | **Sequence (5’-3’)** |
| Human-CIP2A | TGCGGCACTTGGAGGTAATTTC |
|  | AGCTCTACAAGGCAACTCAAGC |
| Mouse-CIP2A | GTGTGAAAGGATGGTCAAAGC |
|  | CCCAGGTCAATCTTGCCATATG |
| Human-RNF4 | GTGACTACCCATACTCCCAGAAACGCC |
|  | GGCTGTCTGTCTGTCCATCCGTCTCTC |
| Human-UBR5 | ACGAGAAGGAAAGCACCATG |
|  | CTTCTCAGAAACTTCTCGTAAC |
| Human-CUL3 | TGTCGAATCTGAGCAAAGGC |
|  | CATCCATGGTCATCGGAAAGG |
| Human-CHIP | AGGCCAAGCACGACAAGTACAT |
|  | CTGATCTTGCCACACAGGTAGT |
| Human-VHL | GTCGAAGAGTACCGCCCTGAAG |
|  | GTGTCCCTGCATCTCTGAAGAG |
| Human-BRCA1 | TTGCGGGAGGAAAATGGGTAGTTA |
|  | TGTGCCAAGGGTGAATGATGAAAG |
| Human-GAPDH | GTCTCCTCTGACTTCAACAGCG  ACCACCCTGTTGCTGTAGCCAA |
| Mouse-GAPDH | AGGTCGGTGTGAACGGATTTG |
|  | TGTAGACCATGTAGTTGAGGTCA |
| **Primers for protein expression plasmid construction** | **Sequence (5’-3’)** |
| pET28a-VHL | GTGCCGCGCGGCAGCCATATGCCCCGGAGGGCGGAGAAC |
|  | GCTTTGTTAGCAGCCGGATCCTCAATCTCCCATCCGTTGATG |
| pET28a-TCEB2 | GTGCCGCGCGGCAGCCATATGGACGTGTTCCTCATGATCCGG  GCTTTGTTAGCAGCCGGATCCTCACTGCACGGCTTGTTCATT |
| pET28a- TCEB1 | GTGCCGCGCGGCAGCCATATGGATGGAGAGGAGAAAACCTATGGT  GCTTTGTTAGCAGCCGGATCCTTAACAATCTAAGAAGTTCGCAGCC |
| pGEX-6P-1-CIP2A (1-560) | TTCCAGGGGCCCCTGGGATCCGACTCCACTGCCTGCTTGAAGT  TCAGTCAGTCACGATGCGGCCGCTCATGTTTCCTGTTGTCTATAGGCA |
| **Primers for overexpression plasmid construction** | **Sequence (5’-3’)** |
| pCDH-Flag-Human-VHL | AAGTAGAGCCCGGGCGGATCCATGCCCCGGAGGGCGGAG  CGATCGCAGATCCTTGCGGCCGCTCAATCTCCCATCCGTTGATG |
| pCDH-Flag-Mouse-VHL | AAGTAGAGCCCGGGCGGATCCATGCCCCGGAAGGCAGCC  CGATCGCAGATCCTTGCGGCCGCTCAAGGCTCCTCTTCCAGGTG |
| pCDH-Flag-Mouse-CIP2A | GAATTCATGGATTCCACCGCCTGCTTG  GCGGCCGCTTATATACTAAGGTTCACAGTTTCTGGGC |
| pCDH-Flag-Human -CIP2A | GAATTCGCCACCATGGACTCCACTGCCTGCTTG  GCGGCCGCCTATATACTGAGATTCACAGTTTCTGGATT |
| pCDH-Human-CIP2A-rescue | AGCTTTTGCGGCACTTGGAAGTAATTTCTGGACAGAAAC  GTTTCTGTCCAGAAATTACTTCCAAGTGCCGCAAAAGCT |
| pCDH-Mouse-CIP2A-rescue-F | CCATTCTGGTGCCCATATCGATGACTTAATTACATTC  AATGTAATTAAGTCATCGATATGGGCACCAGAATGG |

**Table S4:** **Antibody Resource**

| Antibodies | Dilution ratio | Source | Cat# | Application |
| --- | --- | --- | --- | --- |
| PARP | 1:1000 | CST | 9542 | Western Blot |
| PP2Ac | 1:1000 | CST | 2038 | Western Blot |
| AKT | 1:1000 | CST | 9272 | Western Blot |
| p-AKT (Ser473) | 1:1000 | CST | 4060 | Western Blot |
| XPO5 | 1:1000 | CST | 12565 | Western Blot |
| VHL | 1:1000 | CST | 68547 | Western Blot |
| β-actin | 1:1000 | CST | 4970 | Western Blot |
| HA | 1:1000 | CST | 3724S | Western Blot |
| c-Myc | 1:1000 | Abcam | ab32072 | Western Blot |
| CIP2A | 1:200 | Santa Cruz | sc-80659 | Western Blot |
| FLAG | 1:1000 | Sigma-Aldrich | F1804 | Western Blot |
| Ki67 | 1:200 | CST | 9027 | Immunohistochemistry |
| Cleaved Caspase-3 | 1:2000 | CST | 9664 | Immunohistochemistry |

**Supplementary Figures (Figure S1 to Figure S7).**

**
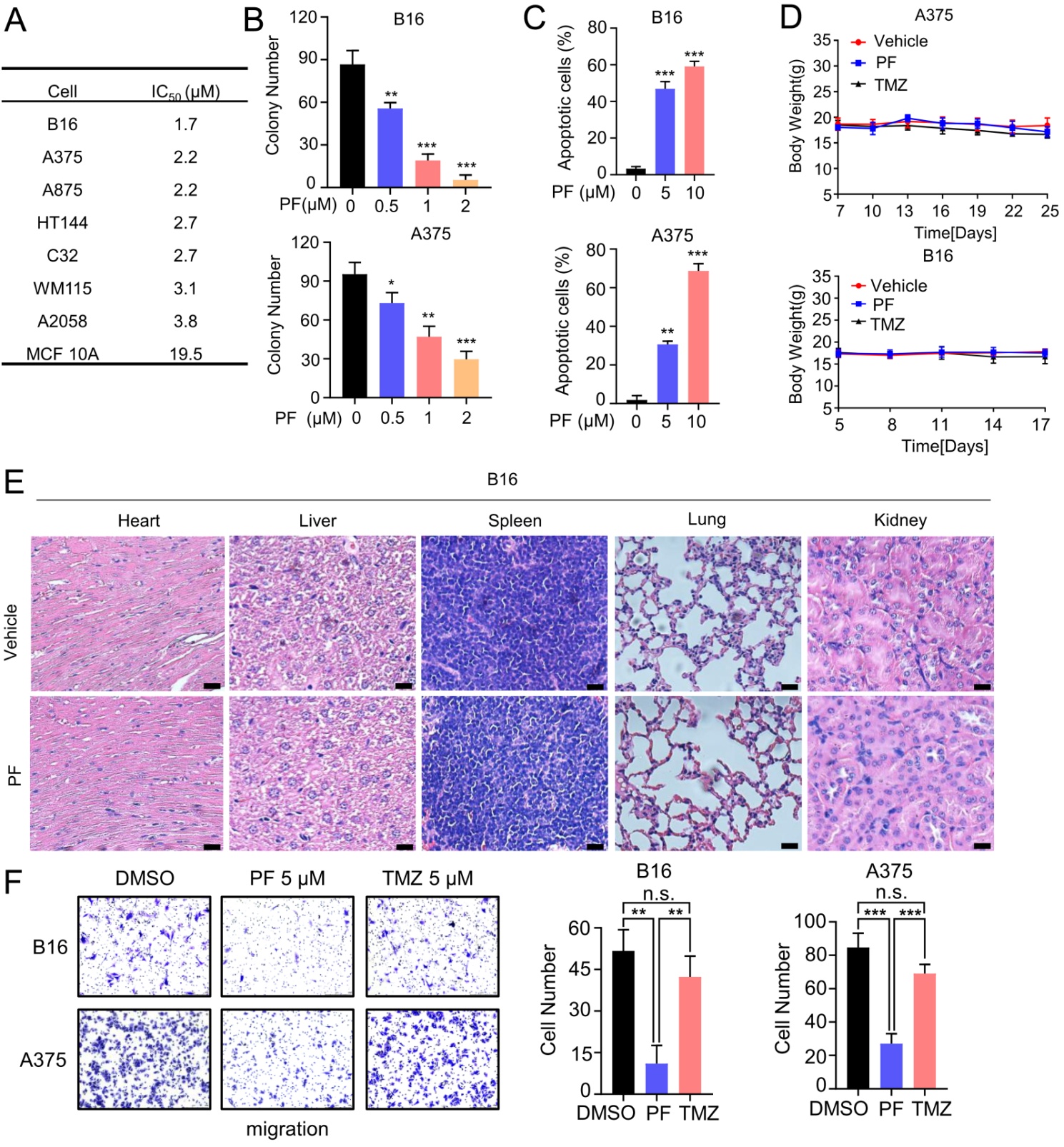
**

**Figure S1. The effects of PF on melanoma cell growth, apoptosis, and migration *in vitro*, as well as its impact on major organs in mice *in vivo*.**

(A) IC_50_ values of PF against seven melanoma cells and MCF 10A cells after 72 h of treatment. (B) Quantification of the results of colony formation assay in Figure 1C. (C) Quantification of the results of apoptosis assay in Figure 1D. (D) Changes in body weight of mice in the two models in Figure 1F and 1G. (E) Hematoxylin and eosin (H&E) stained sections of vital organs from mice treated with either the vehicle or PF. The scale bars represent 50 μm. (F) The effects of 5μM PF and TMZ on the migration of A375 and B16 cells after 24 h of treatment. Data are expressed means ± s.d. **p* < 0.05, ** *p* < 0.01, *** *p* < 0.001.


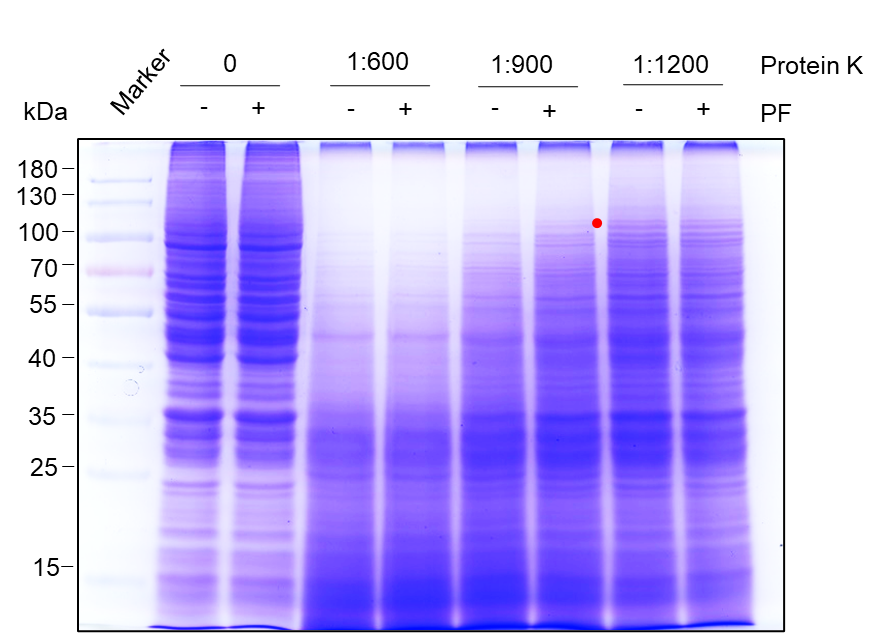


**Figure S2. DARTS assay was performed to detect the effect of PF on the protease K digestion stability of CIP2A protein.** After treating A375 cells with PF (10 μM), whole-cell lysates were treated with different concentrations of protease K for 5 min, followed by SDS-PAGE separation and Coomassie brilliant blue staining. The red dots indicate the difference bands.


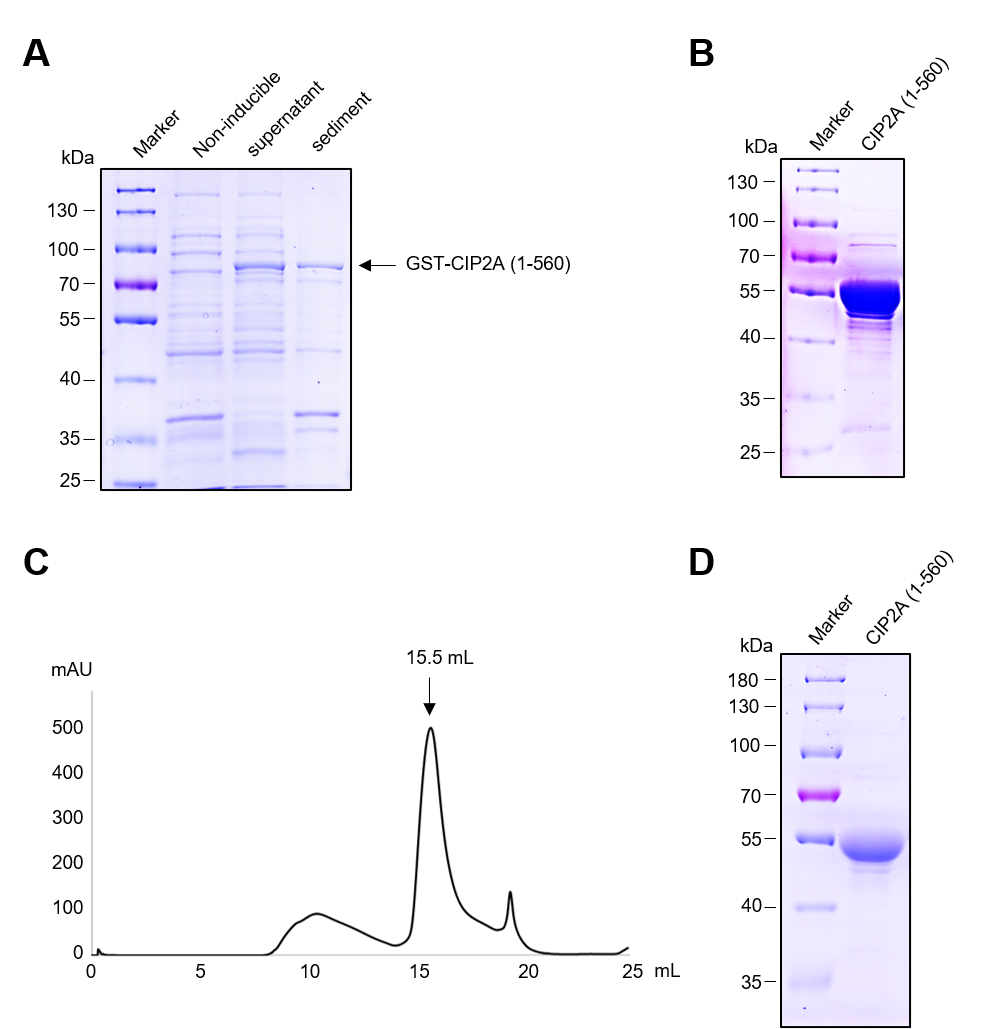


**Figure S3. The expression and purification of CIP2A (1-560).**

(A) SDS-PAGE was showed GST-CIP2A (1-560) expressed in Escherichia coli followed by Coomassie Brilliant Blue staining. (B) SDS-PAGE was showed CIP2A (1-560) after GST affinity chromatography and TEV enzyme cleavage GST followed by Coomassie Brilliant Blue staining. (C) Size-exclusion chromatography purification chromatogram of CIP2A (1-560) after GST affinity chromatography. (D) Coomassie Brilliant Blue staining of the target protein sample from the 15.5 mL peak in (C) after SDS-PAGE.

**
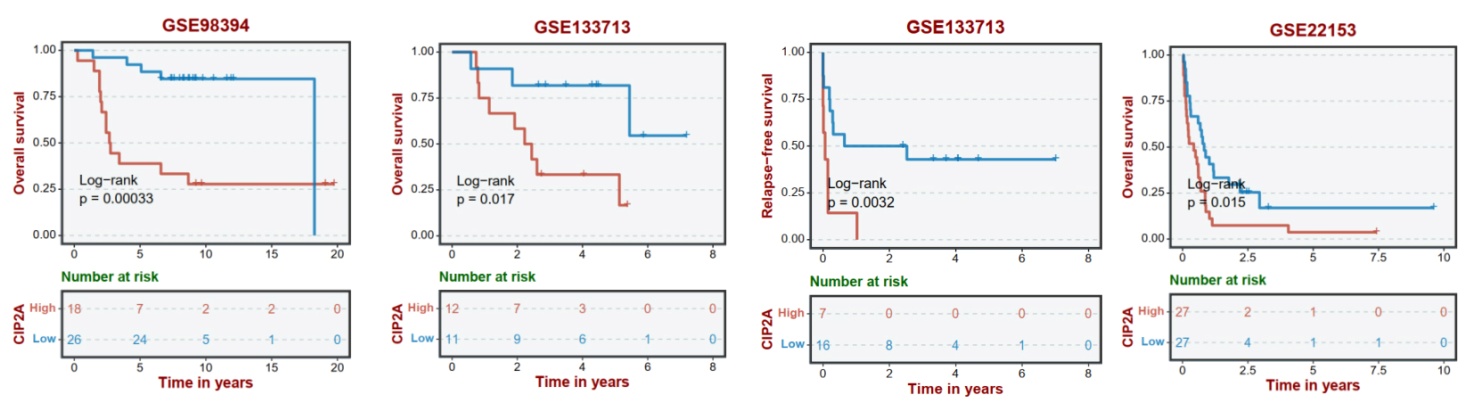
**

**Figure S4. The relationship between CIP2A protein expression and overall survival and relapse-free survival in melanoma patients across several GEO databases.**

**
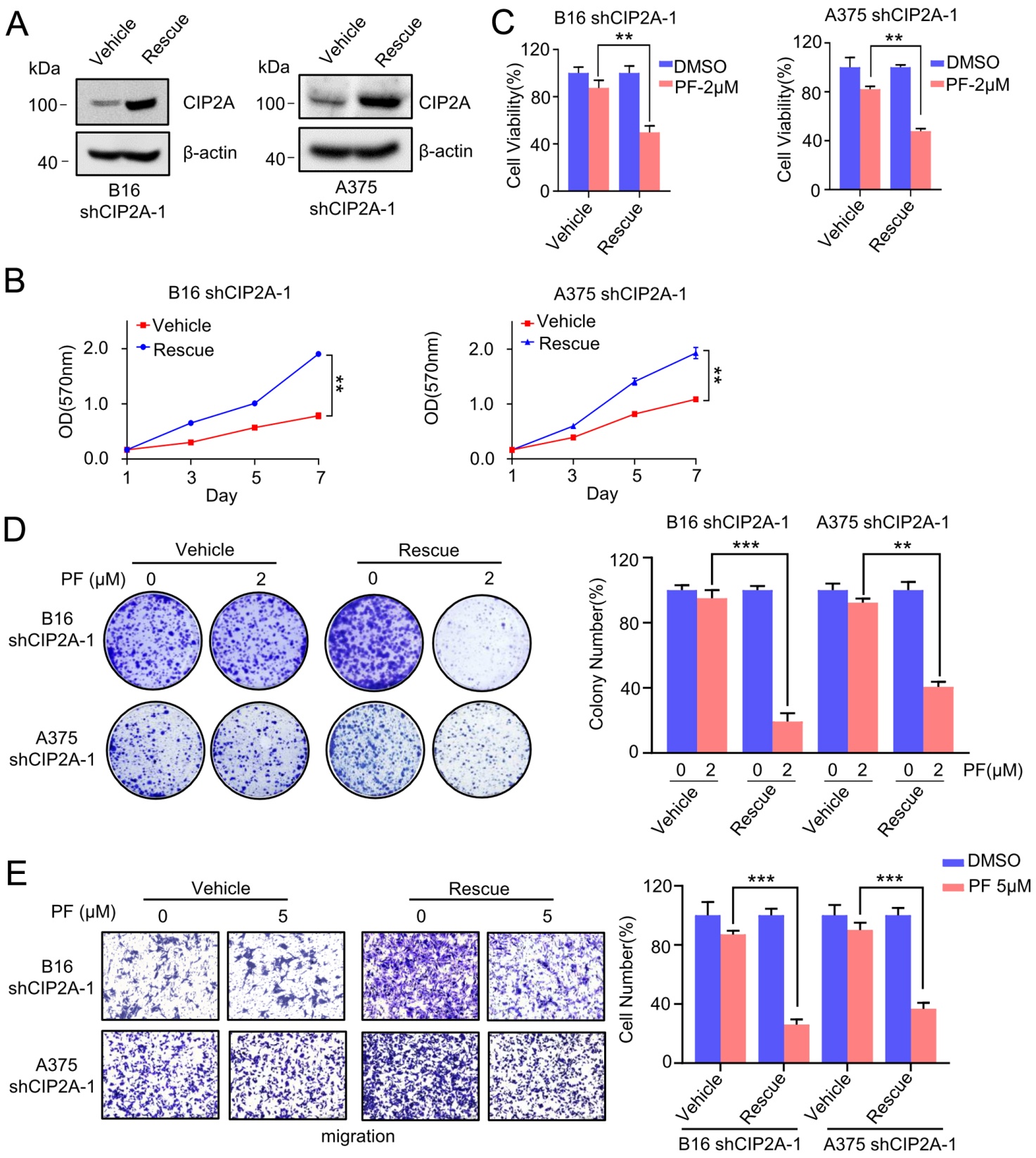
**

**Figure S5. Overexpression of CIP2A enhanced the sensitivity of melanoma cells to PF.**

(A) Western Blot was used to detect the rescue efficiency of CIP2A in B16 and A375 cells with shRNA knockdown of CIP2A. (B) The MTT assay was used to show the effect of CIP2A overexpression on the proliferation of B16 and A375 shCIP2A-1 cells. (C) B16 and A375 shCIP2A-1 cells overexpressing CIP2A were treated with 2 μM PF for 72 h, and the cell viabilities were measured by MTT assay. The viability of cells treated with DMSO in each group was set as 100%. (D) B16 and A375 shCIP2A-1 cells were treated with 2 μM PF for ten days. Then, the impact of CIP2A expression on PF's ability to inhibit cell proliferation was determined by counting the formed colonies. The colony formation rate in each group treated with DMSO was set as 100%. (E) B16 and A375 shCIP2A-1 cells overexpressing CIP2A were treated with PF for 24 h. The effect of CIP2A re-expression on PF's ability to inhibit cell migration was determined by the Transwell migration assay. The migration rate of cells treated with DMSO in each group was set as 100%. Data were presented as means ± s.d. ** *P* < 0.01, *** *P* < 0.001.

**
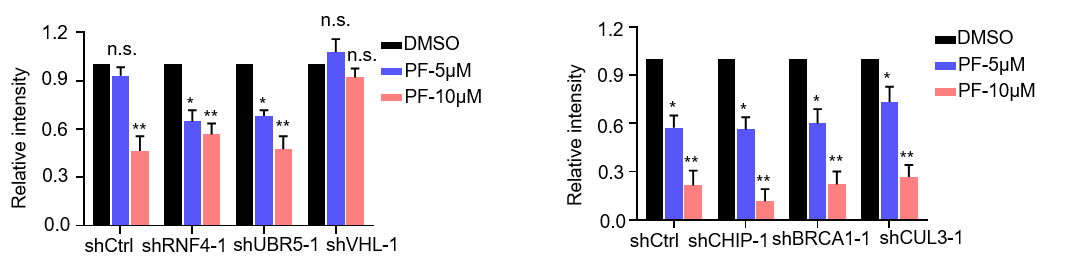
**

**Figure S6. Quantification of the intensity of bands in Figure 6b.** The results are normalized to the untreated conditions (DMSO) in each cell line.


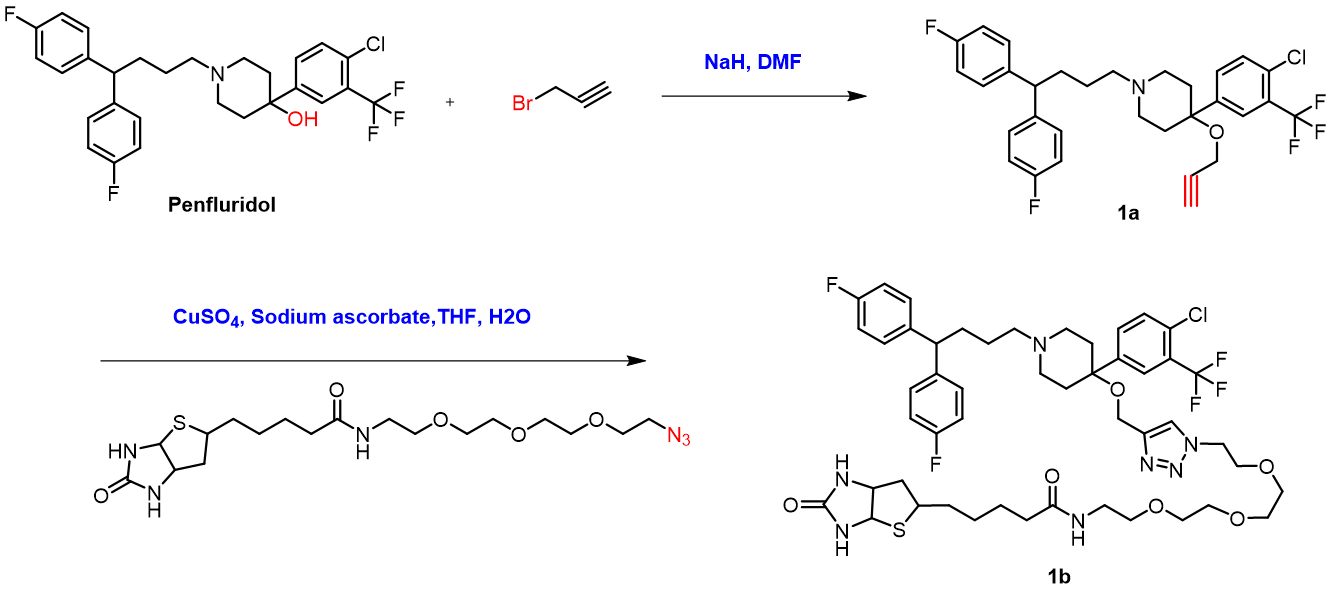
**Figure S7. Design and Chemical synthesis route of PF-Biotin (1b)**

**1-(4,4-bis(4-fluorophenyl)butyl)-4-(4-chloro-3-(trifluoromethyl) phenyl)-4-(prop-2-yn-1-yloxy) piperidine (1a). ^1^H NMR** (400 MHz, chloroform-*d*) δ 7.72 (d, *J* = 15.9 Hz, 1H), 7.55 – 7.46 (m, 2H), 7.16 (dd, *J* = 8.6, 5.4 Hz, 4H), 6.96 (t, *J* = 8.7 Hz, 4H), 5.15 (d, *J* = 5.9 Hz, 1H), 3.88 (t, *J* = 7.8 Hz, 1H), 3.75 (d, *J* = 2.5 Hz, 1H), 2.81 (d, *J* = 9.0 Hz, 2H), 2.61 – 2.44 (m, 4H), 2.39 (t, *J* = 2.4 Hz, 1H), 2.13 (d, *J* = 8.0 Hz, 2H), 2.07 – 1.97 (m, 4H), 1.54 (q, *J* = 8.9, 7.7 Hz, 2H). **^13^C NMR** (100 MHz, chloroform-*d*) δ 202.77, 162.61, 160.18, 140.35, 131.83, 131.65, 130.48, 129.12, 129.04, 125.32, 125.27, 125.22, 115.44, 115.23, 114.03, 88.04, 79.91, 74.16, 58.15, 51.17, 49.70, 49.67, 48.96, 48.74, 34.53, 33.73. **HR-ESIMS**: 562.1922 [M+H]^+^ (calc. for C_31_H_29_ClF_5_NO, 562.1931).


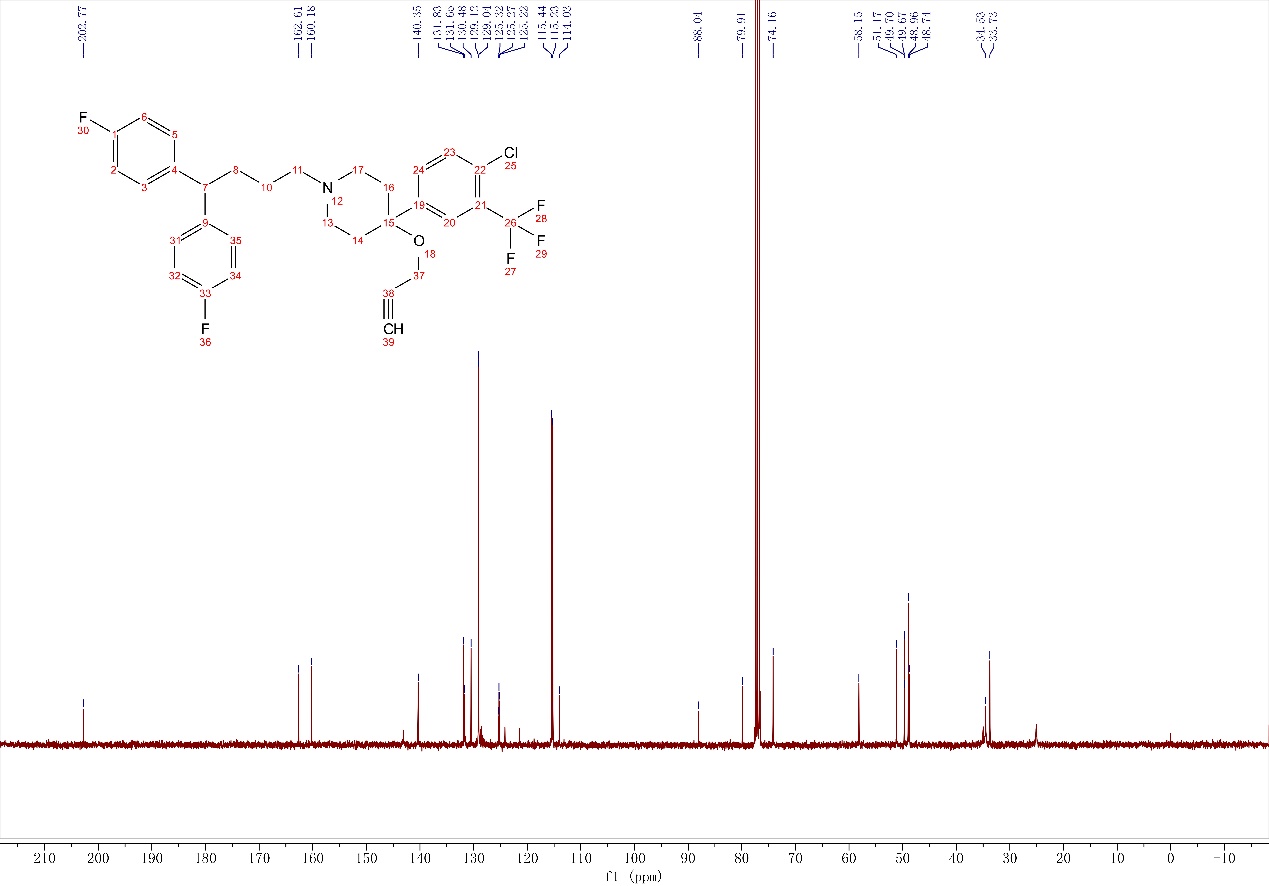

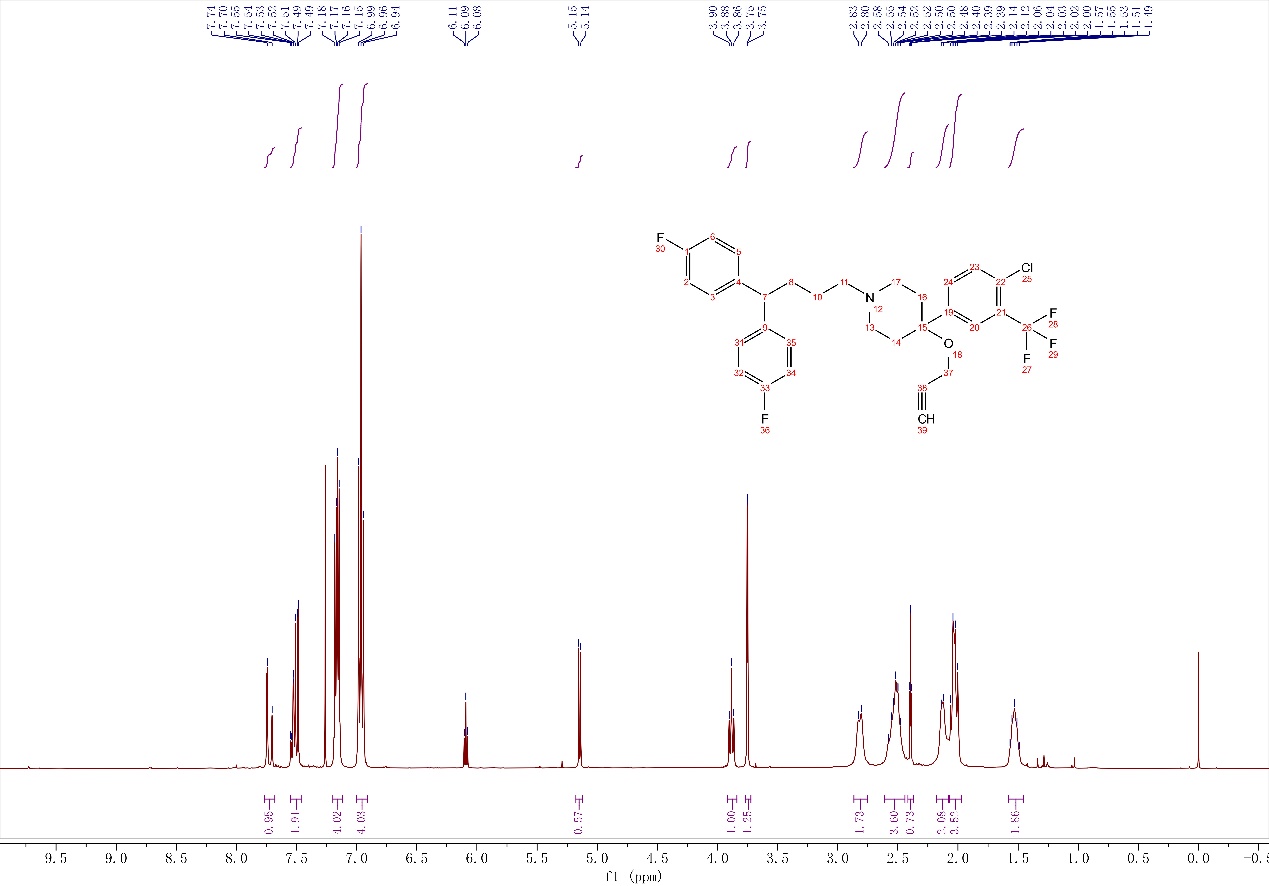

**N-(2-(2-(2-(2-(4-(((1-(4,4-bis(4-fluorophenyl)butyl)-4-(4-chloro-3-(trifluoromethyl) phenyl) piperidin-4-yl)oxy)methyl)-1H-1,2,3-triazol-1-yl)ethoxy)ethoxy)ethoxy)ethyl)-5-(2-oxohexahydro-1H-thieno[2,3-d]imidazol-5-yl)pentanamide (1b, PF-Biotin). ^1^H NMR** (400 MHz, DMSO-*d*_6_) δ 7.99 (s, 1H), 7.86 (s, 1H), 7.80 (d, *J* = 9.5 Hz, 2H), 7.74 (d, *J* = 8.4 Hz, 1H), 7.33 (dd, *J* = 8.6, 5.7 Hz, 4H), 7.10 (t, *J* = 8.8 Hz, 4H), 6.39 (d, *J* = 22.9 Hz, 2H), 4.51 (t, *J* = 5.2 Hz, 2H), 4.30 (dd, *J* = 7.7, 5.0 Hz, 1H), 4.11 (s, 3H), 3.99 (t, *J* = 7.9 Hz, 1H), 3.82 (t, *J* = 5.2 Hz, 2H), 3.53 (dd, *J* = 6.1, 3.4 Hz, 2H), 3.51 – 3.48 (m, 2H), 3.47 (s, 4H), 3.36 (d, *J* = 6.0 Hz, 4H), 3.17 (q, *J* = 5.9 Hz, 2H), 3.08 (dt, *J* = 8.7, 5.6 Hz, 1H), 2.81 (dd, *J* = 12.4, 5.1 Hz, 1H), 2.59 (t, *J* = 11.6 Hz, 3H), 2.28 (dt, *J* = 27.5, 8.9 Hz, 4H), 2.09 – 1.87 (m, 8H), 1.61 (ddt, *J* = 12.4, 9.4, 6.2 Hz, 1H), 1.47 (ddt, *J* = 22.1, 13.8, 7.9 Hz, 3H), 1.38 – 1.25 (m, 4H). **^13^C NMR** (100 MHz, DMSO-*d*_6_) δ 172.57, 163.18, 162.28, 159.88, 145.49, 144.37, 141.78, 141.75, 132.45, 132.22, 130.00, 129.78, 129.70, 127.23, 126.92, 125.83, 125.78, 124.76, 124.36, 122.04, 115.64, 115.43, 76.12, 70.16, 70.11, 70.01, 69.63, 69.18, 61.50, 59.67, 57.97, 56.18, 55.89, 49.78, 49.20, 49.17, 38.89, 35.56, 34.91, 33.33, 28.66, 28.50, 25.72, 25.61. **HR-ESIMS**: 1006.4119 [M+H]^+^ (calc. for C_31_H_29_ClF_5_NO, 1006.4085).

**
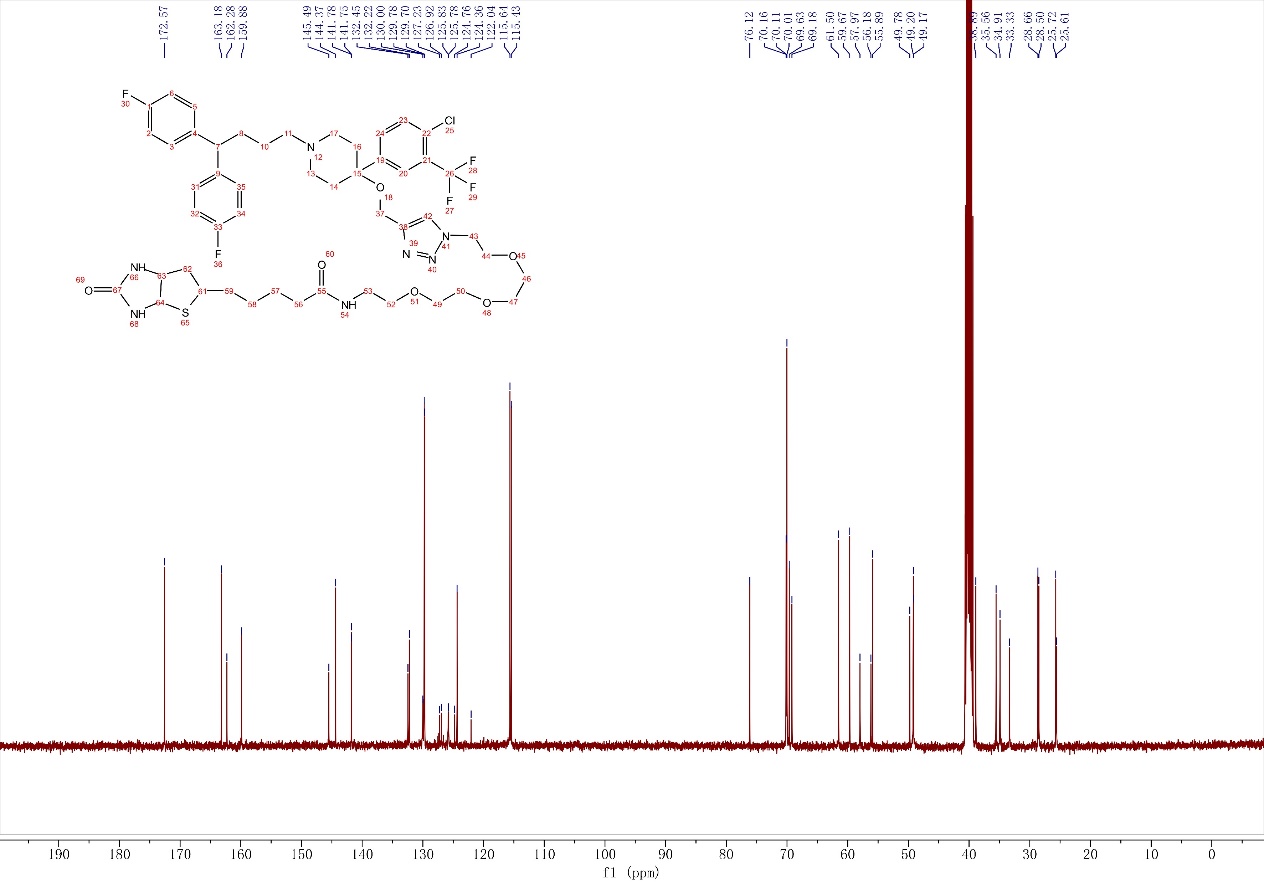

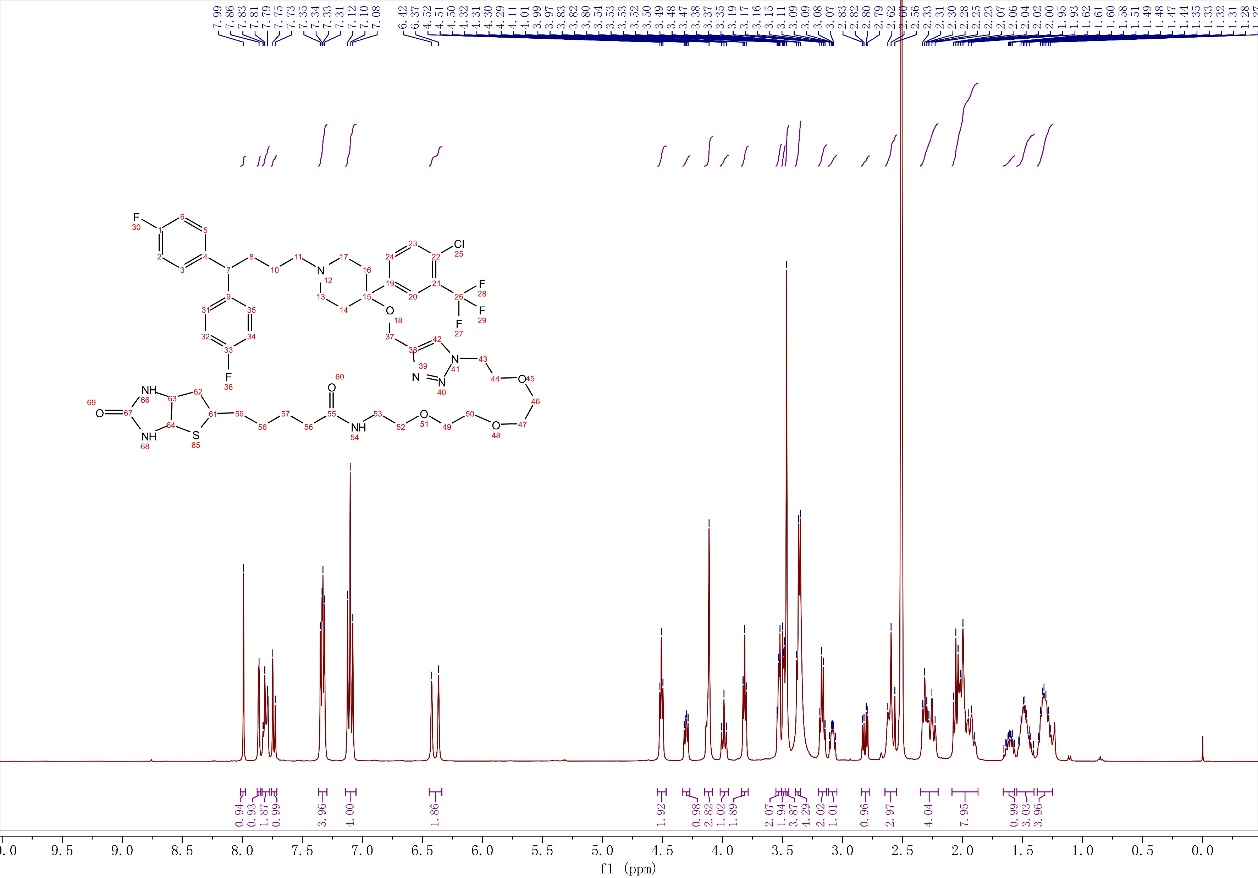
**

**
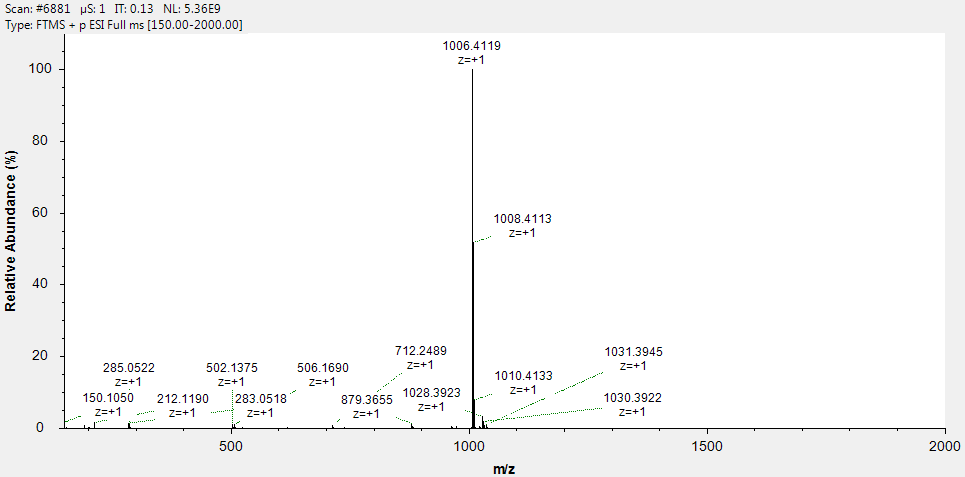
**
